# Glioblastoma antitumoral activity of tetrahydroquinoline-derived triarylmethanes

**DOI:** 10.1039/d5md00585j

**Published:** 2025-10-24

**Authors:** Daniela S. N. Branco, Zahra Hosseinpur Yektaei, Sureka Chandrabose, Filipe A. Almeida Paz, Meenakshisundaram Kandhavelu, Nuno R. Candeias

**Affiliations:** a LAQV-REQUIMTE, Department of Chemistry, University of Aveiro 3810-193 Aveiro Portugal ncandeias@ua.pt; b Molecular Signaling Group, Faculty of Medicine and Health Technology, Tampere University Tampere Finland meenakshisundaram.kandhavelu@tuni.fi; c BioMeditech and Tays Cancer Center, Tampere University Hospital, P.O. Box 553 33101 Tampere Finland; d Department of Basic Medical Sciences, College of Medicine, Prince Sattam Bin Abdulaziz University Al-Kharj 11942 Saudi Arabia; e CICECO – Aveiro Institute of Materials, Department of Chemistry, University of Aveiro Campus Universitário de Santiago Aveiro 3810-193 Portugal; f Faculty of Engineering and Natural Sciences, Tampere University Korkeakoulunkatu 8 33101 Tampere Finland

## Abstract

Glioblastoma multiforme (GBM) is an aggressive and treatment-resistant brain tumor. The expansion of a phenolic Mannich base library *via* the Petasis reaction unexpectedly led to the unsymmetrical tetrahydroquinoline-derived triarylmethanes, confirmed by single-crystal X-ray diffraction. Optimization of reaction conditions revealed the influence of solvent, temperature, and substituent patterns on product yield and regioselectivity. Several of the newly synthesized triarylmethanes demonstrated potent cytotoxicity against human GBM cell lines LN229 and SNB19, with compound 8a′ exhibiting IC_50_ values (35.3 μM and 23.5 μM, respectively) significantly lower than those of the standard chemotherapeutic agent temozolomide (309.7 μM and 344.4 μM, respectively). In addition to inhibiting cell proliferation, 8a′ disrupted GBM cell migration in scratch assays, suggesting a strong link between cytotoxicity and impaired motility. The SiRNA experiment confirmed that the specific interaction of 8a′ with EGFR modulates intracellular calcium levels in GBM. These findings highlight the therapeutic potential of triarylmethane scaffolds in GBM treatment *via* EGFR interaction and underscore the importance of fine-tuning multicomponent reactions to discover biologically active chemotypes.

## Abstract 2

Glioblastoma multiforme (GBM) is an aggressive and treatment-resistant brain tumor.

## Graphical abstract

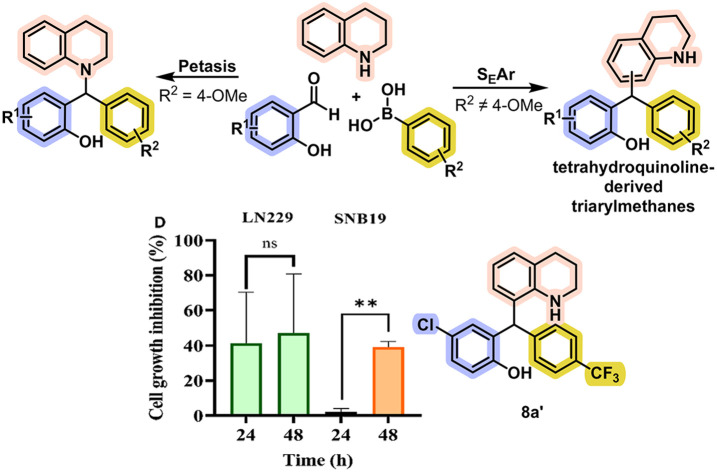

## 1. Introduction

Glioblastoma multiforme (GBM) is a grade IV glial tumor with a poor prognosis. Its high aggressiveness, rapid proliferation, and tendency to infiltrate healthy brain tissue make this tumor one of the most challenging cancers in oncology.^1^ According to epidemiological data from the Central Brain Tumor Registry of the United States (CBTRUS), GBM was in 2020 the most common malignant tumor affecting the central nervous system (CNS), accounting for 49% of all malignant CNS tumors and 15% of all brain cancers.^2^ This pathology has an annual incidence of 3.1 per 100 000 individuals,^3,4^ recently predicted to increase by almost 50% in the upcoming 30 years.^5^ The average GBM survival time is twelve to fifteen months, with a 5-year survival rate of 7.2%. Despite the aggressive standard treatment of GBM, maximal surgical resection followed by chemoradiotherapy using temozolomide (TMZ), GBM tumors tend to recur after treatment.^6^ Moreover, the self-renewing capabilities and robust DNA repair of glioblastoma cells lead to resistance against currently available treatments.^7^ Despite the several cytotoxic and anti-angiogenic chemotherapeutic agents developed, the genomic complexity and multiple signaling pathways of GBM demand the development of new chemotherapeutic agents.^8^

Some years ago, impelled by the availability of a considerable-sized library of phenolic Mannich bases (pMb), we embarked on a medicinal chemistry program for the evaluation of such molecules as cytotoxic agents for different tumors. pMb Include a wide range of biological activities and various subclasses, depending on the substrates used.^9,10^ They can be described as a prodrug activated by pH changes, resulting in a deamination product, known as *ortho*-quinone methide.^11^ This reactive and electrophilic species acts as an alkylating agent for thiols and amine groups from biomolecules.^12,13^

**Scheme 1:**
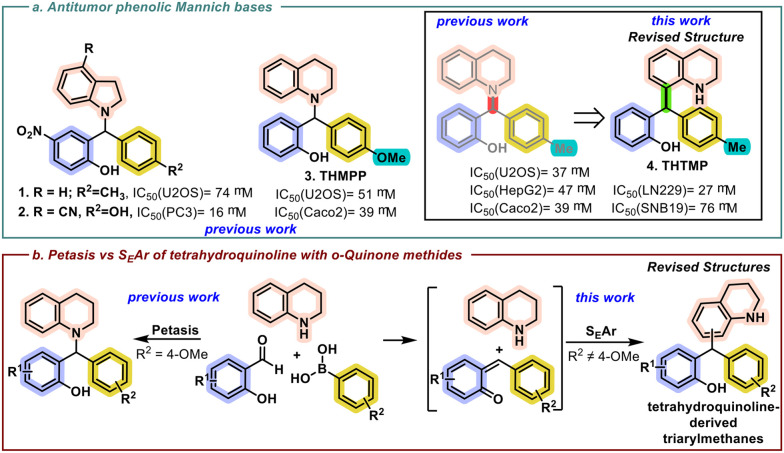
a. Previously reported antitumor activity of pMb and structure revision of tetrahydroquinoline derivative; b. expansion of tetrahydroquinoline-derived triarylmethane library and revision of previous structures (R^1^ = H, R^2^ = Me, 4-Ph, 4-Cl, 4-CF_3_; R^1^ = NO_2_, R^2^ = 4-Cl).^22^

pMb Can be efficiently prepared using a multicomponent approach known as Petasis reaction^14,15^ combining salicylaldehyde derivatives, an amine, and an aryl boronic acid. Such a procedure has enabled us to synthesize various heterocyclic derivatives, exploring their antitumor properties. Specifically, morpholine and indoline-derived pMb are cytotoxic against osteosarcoma cell lines,^16^ indoline derivatives being also effective against prostate cancer^17^ and GBM.^18^ During our studies (Scheme 1a), tetrahydroquinoline was also explored as a Petasis reaction component, allowing the identification of methoxy-substituted pMb, 2-((3,4-dihydroquinolin-1(2*H*)-yl)(4-methoxyphenyl)methyl)phenol, (**THMPP**) as a promising cytotoxic agent against osteosarcoma,^19^ glioblastoma^20^ and colon cancer.^21^ Replacing the methoxy with a methyl substituent on the aromatic moiety of the boronic acid resulted in the synthesis of an unsymmetrical triarylmethane, 2-((1,2,3,4-tetrahydroquinolin-8-yl)(*p*-tolyl)methyl)phenol (**THTMP**). Regardless the obtention of the unanticipated triarylmethane instead of pMb, **THTMP** presents a positive effect at GBM cytotoxicity level.^20^ Our investigation of cellular mechanisms reveals that **THTMP** treatment disrupts epidermal growth factor receptor (EGFR) pathways by modulation of genes encoding downstream mediators,^23^ leading to a reduction of the cell proliferation and migration. Further tetrahydroquinoline derivatives have been reported to inhibit key signalling pathways and proteins, such as receptor tyrosine kinases and NF-κB, which are crucial for cancer cell proliferation and survival.^24^ The structural versatility of tetrahydroquinolines allows for the design of novel compounds with enhanced anticancer properties, making them a focal point in the development of new therapeutic agents.^25^

Pushed by the interesting cytotoxic properties associated with the tetrahydroquinoline moiety, such a library was recently expanded to consider different substituent patterns in the other two aryl rings.^22^ During the continuation of our studies on the Petasis reaction, we have identified the occurrence of an alternate reaction pathway resulting in the formation of unsymmetrical triarylmethanes (Scheme 1b). Herein, in light of these new findings, and based on crystal X-ray diffraction analysis, we are reporting the expansion of the library of tetrahydroquinolines and the revision of the previously reported structures^22^ (Scheme 1b).

Triarylmethanes (TAMs) are a privileged scaffold in medicinal chemistry,^26–32^ explored multiple times for the development of antitumor agents. *S*-Trityl l-cysteine derivatives have been reported as potent anticancer agents of NCI 60 tumor cell line, by arresting cells' mitotisis.^33^ Clotrimazole and other TAM derivatives have been explored as antitumoral agents, identified to arrest the cell cycle in G0–G1 phase.^34^ Indole-^35^ and oxindole-derived^36^ TAMs have also been multiply explored for their anti-tumor properties, and other heterocyclic-derived TAMs were explored as anti-breast cancer agents.^37^ More recently, TAMs have been explored in the development of anti-colorectal cancer agents, of which pyridine *N*-oxides were particularly antiproliferative agents,^38^ and as agonists for aryl hydrocarbon receptor,^39^ a target for cancer treatment.^40^

## 2. Results and discussion

**Fig. 1:**
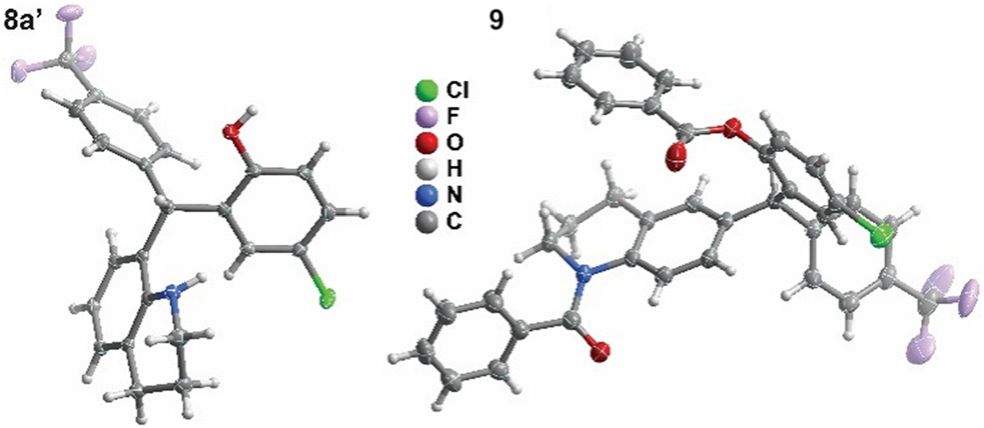
X-ray diffraction of 8a′ and 9. Thermal ellipsoids are shown at the 50% probability level; hydrogen atoms are shown with an arbitrary radius (0.30 Å).

During our actions to expand the library of tetrahydroquinoline derivatives, we set out to explore the importance of the salicylaldehyde substituent in the antitumoral properties, blocking the presence of the *p*-CF_3_ substituent provenient from the arylboronic acid. When using the previously established reaction conditions of refluxing a mixture of salicylaldehyde 5a, 4-(trifluoromethyl)phenylboronic acid 6 and 2 equivalents of tetrahydroquinoline 7 in toluene, led to the formation of two isomeric species of almost identical ^1^H and ^13^C NMR traces and mass analysis. The structure of the isomers was elucidated by X-ray diffraction to reveal the formation of triarylmethanes 8a′ and 8a″ (Table 1, entry 1). The former was crystallized as such, while the latter required its derivatization to the dibenzoylated congener 9 (Fig. 1). Attempts to improve the yield and selectivity of the transformation were made by screening the stoichiometric ratios of the reactants and reaction solvent (Table 1). Decreasing the excess of the amine and boronic acid had a detrimental effect on the yield, despite the unchanged isomeric ratio (entries 2–3). A high excess of the amine kept the yield and selectivity unchanged (entry 4). Lowering the reaction temperature required extending the reaction time, resulting in the formation of the isomers in 27% yield after 31 h, although without a noticeable effect on the isomeric ratio (entry 5).

**Table 1:** Screening conditions for formation of tetrahydroquinoline-derived triarylmethanes

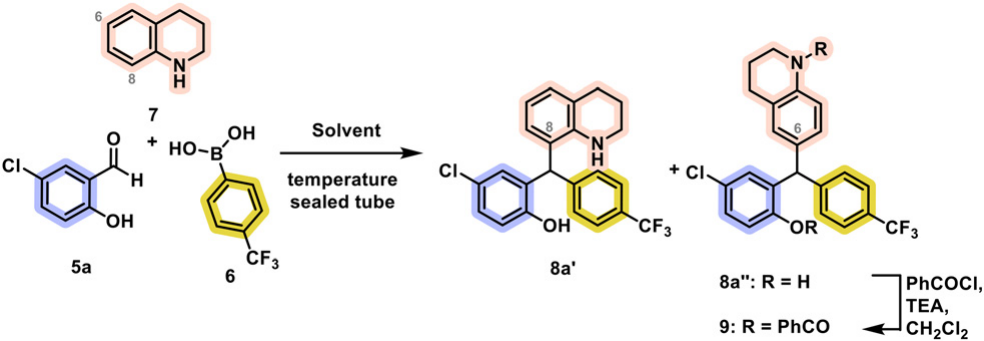							
Entry^a^	5a : 6 : 7 ratio	[5a] (M)	Solvent	Temp (°C)	Reaction time (h)	Yield^b^ (%)	8a′ : 8a″ ratio^b^
1	1 : 1.2 : 2	0.1	Toluene	110	3	49	78 : 22
2	1 : 1 : 1	0.1	Toluene	110	3	33	75 : 25
3	1.5 : 1 : 1	0.15	Toluene	110	3	33	76 : 24
4	1 : 1.2 : 4	0.1	Toluene	110	1.5	41	74 : 26
5	1 : 1.2 : 2	0.1	Toluene	40	31	27	77 : 23
6	1 : 1.2 : 2	0.1	DCE	100	1	40	71 : 29
7	1 : 1.2 : 2	0.1	HFIP	40	20.5	24	24 : 76
8	1 : 1.2 : 2	0.1	ACN	100	3.5	17	37 : 63
9	1 : 1.2 : 2	0.2	Toluene	100	1.5	59	69 : 31

a Reaction performed using 0.1 mmol of aldehyde, followed by the addition of boronic acid and tetrahydroquinoline, and heated at reflux.

b Yield and isomeric ratios were determined through ^19^F NMR, using C_6_F_6_ as an internal standard.

Changing the reaction solvent to boiling dichloroethane had little impact on reaction yields and selectivity (entry 6), but the use of hexafluoroisopropanol or acetonitrile significantly affected the isomeric ratio. Notwithstanding the lower yields, the preferential formation of tetrahydroquinoline derivatives substituted at position 6 (entries 7 and 8) was noticeable. Increasing the concentration of the starting aldehyde to 0.2 M in toluene resulted in an increased yield of 59%, at some small expense of the selectivity (entry 9). Similar conditions were applied to the reaction starting from the pinacol-derived boronic ester of 5a. Interestingly, whilst complete consumption of the aldehyde was observed for every entry in Table 1, refluxing the boronic ester in toluene for a week did not show consumption of the aldehyde, nor formation of any product.

**Scheme 2:**
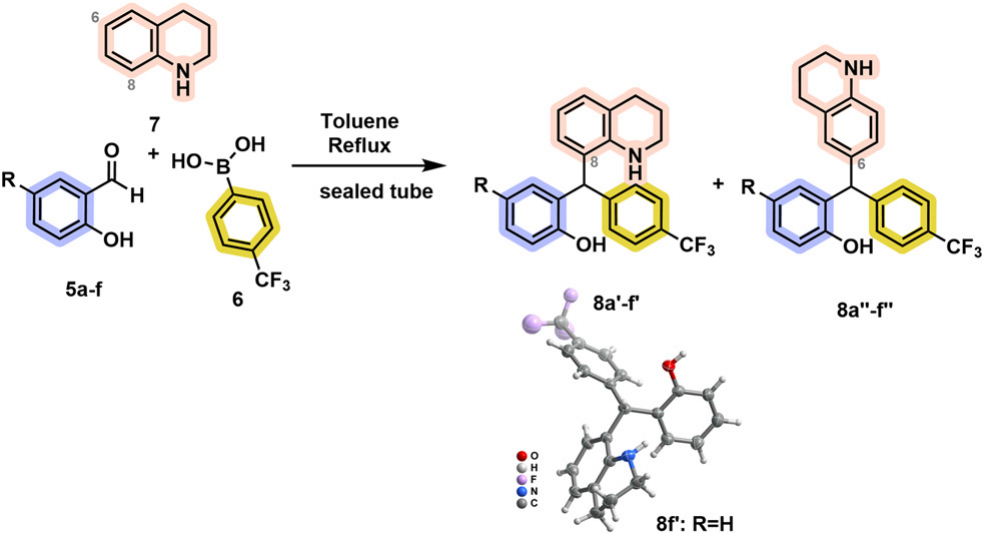
Expansion of TAMs library. Inset shows single-crystal X-ray diffraction of 8f′. Thermal ellipsoids are shown at the 50% probability level.

The generality of the transformation for tetrahydroquinoline was further verified for the unsubstituted salicylaldehyde and phenylboronic acid (see SI, Table S1). Interestingly, when using mild conditions such as dichloromethane at room temperature in the presence of sieves, the formation of the corresponding TAM was achieved in a reasonable 49% yield after 48 h. Upon the structure identification of TAMs 8a′ and 8a″, other derivatives were prepared by replacing the salicylaldehyde counterpart (Scheme 2, Table 2). The reaction success was shown independent of the 5-substituent of the salicylaldehyde, resulting in the formation of products 8′ and 8″ in similar yields, but in slightly better ratios towards the 8-substituted tetrahydroquinoline derivative. The 4-(trifluoromethyl)phenylboronic acid-derived lead compound of our previous study (8f′),^22^*i.e.* from unsubstituted salicylaldehyde, was crystallized and analyzed by X-ray diffraction to confirm the TAM structural framework (Scheme 2, inset). This finding led to a revision of all previously published structures, identifying the TAM rather than pMb. Considering that, the TAM formation is compatible with different substituents in the arylboronic acid moiety in different positions, providing the corresponding TAM in 42–66% yields as previously assessed for methyl substituent at different positions of the aryl ring, and chlorine or phenyl at the 4-position.^22^ On the other hand, the established reaction conditions were not compatible with heteroaromatic boronic acids.

**Table 2:** Expansion of TAMs library

Entry^a^	R	5	Reaction time (h)	Yield^b^ (%)	8′ : 8″ ratio^b^	8′ isolated yield^c^ (%)
1	Cl	5a	1.5	59	69 : 31	22 (3)^d^
2	Br	5b	1.5	68	76 : 24	32
3	F	5c	1	68	81 : 19	41
4	Me	5d	1.5	70	88 : 12	44
5	OMe	5e	4	61	85 : 15	41
6	H	5f	1	51	86 : 14	61

a Reaction performed using 0.1 mmol of aldehyde, followed by the addition of boronic acid and tetrahydroquinoline, and heated at reflux.

b Yield and isomeric ratios were determined through ^19^F NMR, using C_6_F_6_ as an internal standard.

c Reaction performed at 0.5 mmol scale of aldehyde.

d In parentheses is shown the isolated yield for 8a″.

The unexpected failure of the Petasis reaction for the coupling of tetrahydroquinoline can be explained by its electron-rich nature and the intermediacy of *o*-quinone methides (*o*-QM). Such a reaction, in which the Petasis product derived from piperidine engages in a putative formation of *o*-QM followed by electrophilic aromatic substitution of an aromatic amine, has been previously reported to be catalyzed by I_2_ ^41^ or FeCl_3_ ^42^ as Lewis acids. While piperidine requires a catalyst to generate the electrophilic *o*-QM for Petasis product formation, the tertiary amine derived from tetrahydroquinoline undergoes C–N bond cleavage and subsequent C–C bond formation without a catalyst at elevated temperatures. A mechanism based on the intermediacy of *o*-QM is proposed, although the higher propensity towards substitution of tetrahydroquinoline in the 8-position, rather than the 6-position, could be explained by the prevalence of an intramolecular process. During the preparation of this work, Hazra and co-workers have shown the influence of the solvent on the *N*-alkylation *vs. C*-alkylation of arylamines using *o*-QM as alkylating agents.^43^ While toluene was shown to favor the *N*-alkylation, we were not able to detect any of the Petasis products under such conditions when using tetrahydroquinoline.

In line with our previous studies on the biological activity of phenolic Mannich bases, and the recently reported anti-glioblastoma activity of triarylmethanes,^22^ we proceeded to evaluate the biological activity of this newly expanded library of triarylmethanes derived from 4-(trifluoromethyl)phenylboronic acid.

### Tetrahydroquinoline-derived triarylmethanes induce GBM cell death

**Fig. 2:**
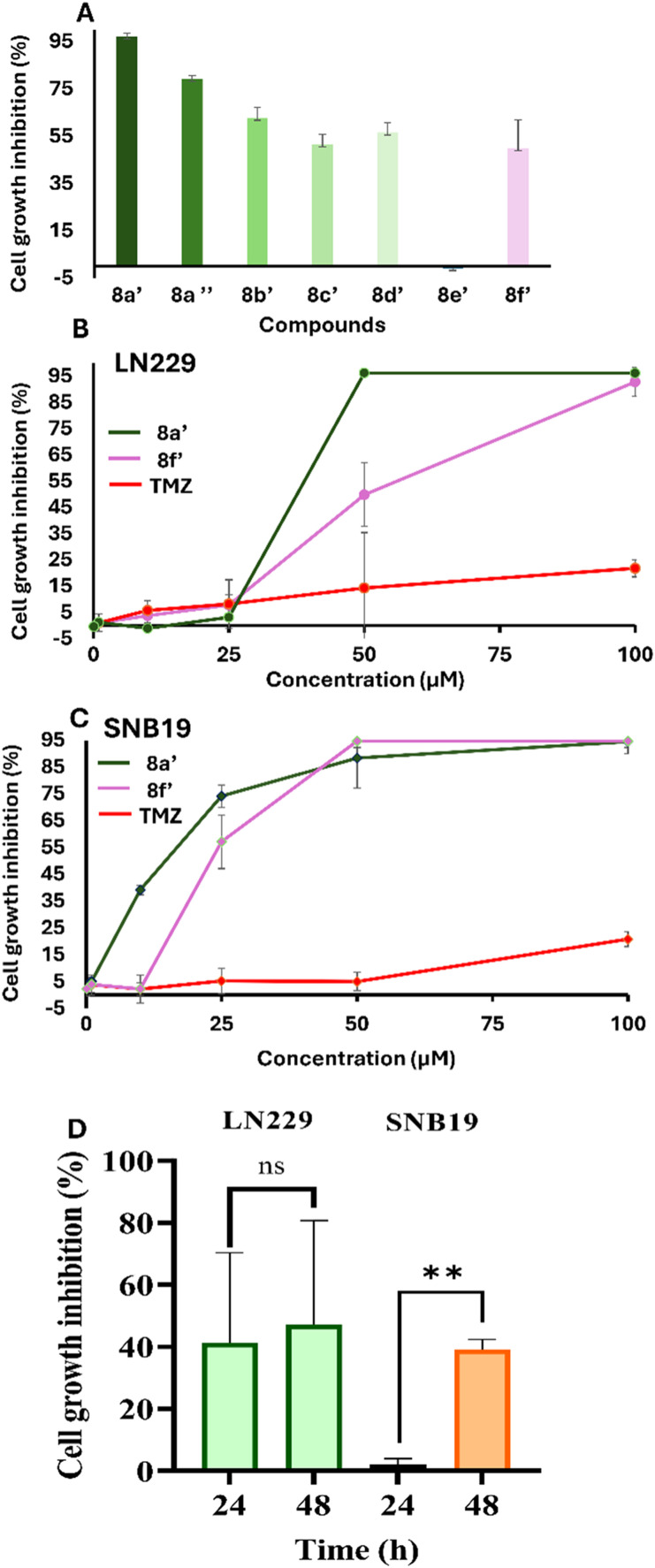
The cytotoxic impacts of tetrahydroquinoline-derived triarylmethanes in GBM cells. A) The percentage of cell growth inhibition of seven triarylmethanes was assessed against the LN229 cell line at a concentration of 50 μM using Trypan blue assay. B) The dose-dependent cytotoxic efficacy of 8a′ was examined on both LN229 and C) SNB19 cell lines. The percentage of cell growth inhibition data normalized against DMSO (mean ± S.D., *n* = 5). D) The time-dependent effect of 8a′ over the 24 hours and 48 hours in LN229 and SNB19 cells. * indicates statistically significant differences between derivative-treated samples and the DMSO, ** *p* < 0.001.

To investigate the cytotoxic effects of newly synthesized tetrahydroquinoline-derived triarylmethanes, the human GBM cell line, LN229, was treated with 50 μM concentration, as described in the methods section. Analysis of cell viability (Fig. 2A) revealed that treatment with compounds 8b′, 8c′, 8d′ and 8f′ resulted in a cytotoxicity rate ranging from 50% to 60%. In contrast, cells incubated with compound 8a′ and 8a″ exhibited significantly higher cytotoxicity of 97% and 79% dead cells, respectively. The presence of the chlorine atom in the phenolic ring was seen to be beneficial for increasing the cytotoxic effect of the triarylmethanes. Replacing chlorine with bromine was not as effective, and adding fluorine had no effect in comparison with the unsubstituted derivative 8f′. The introduction of a methoxy group led to non-cytotoxic compound 8e′, at the concentration tested. Luckily, when comparing the substitution in the tetrahydroquinoline ring, the preferred formed regioisomer 8a′ was determined to be more cytotoxic than its isomer substituted in position 6. Given the most cytotoxicity effect compared to the other derivatives, 8a′ has been identified as a potent candidate for inducing cell death in LN229 cells and was selected for further analysis. In addition, the structurally close related compound 8f′ was also tested along with TMZ control. To evaluate the half-maximal inhibitory concentration (IC_50_) against the proliferation of GBM cells, LN229 and SNB19 were treated with different concentrations of 8a′, 8f′, TMZ and DMSO. After 48 h of incubation, the concentration-dependent inhibition of cell growth was observed for all the compounds. The derivative 8a′ exhibited the IC_50_ of a 35.3 μM and 23.5 μM in LN229 (Fig. 2B) and SNB19 (Fig. 2C), respectively. Compound 8f′ and TMZ demonstrated IC_50_ values of 40.6 μM and 309.7 μM in LN229, while these compounds exhibited IC_50_ values of 38.3 μM and 344.4 μM in SNB19, respectively. To further elucidate the time-dependent effects of compound 8a′ on cellular viability (Fig. 2D), GBM cells were subjected to treatment for a period of 24 and 48 h. 8a′ Showed a 44% and 47% reduction in cell proliferation at 24 h in LN229 cells, while SNB19 cells showed a significant decrease of 3% and 40%, respectively.

### 8a′ mediated cell death affected the cell migration

**Fig. 3:**
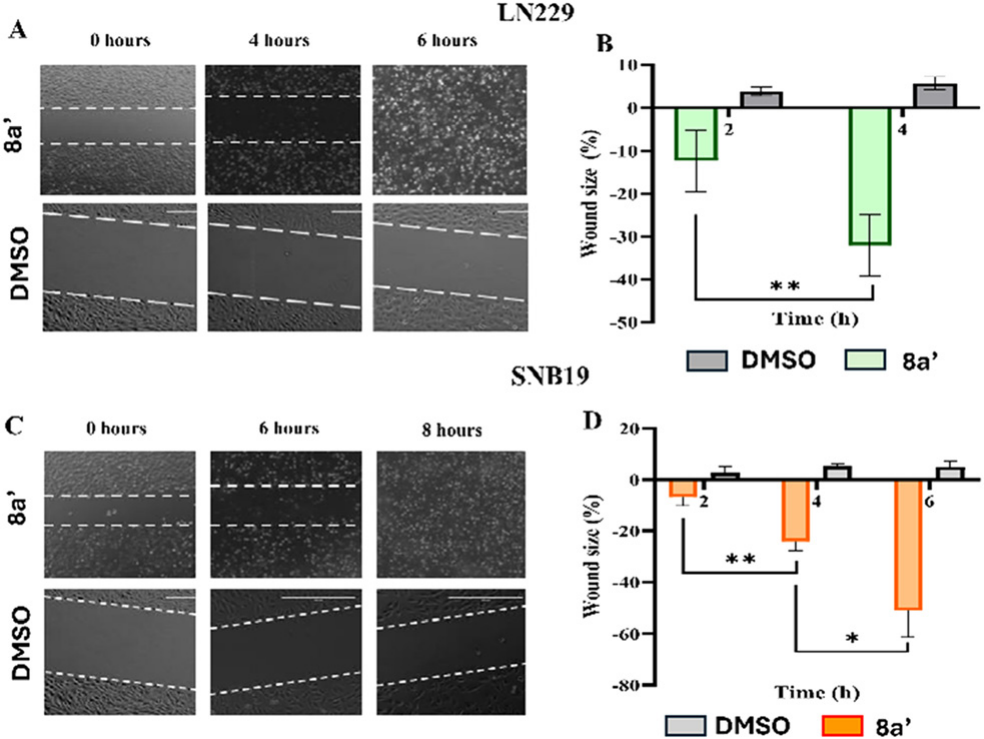
The effect of a novel derivative 8a′ on GBM cell migration. A) Confocal microscopy images of the LN229 cells treated with 8a′ over a 0–8 hour period. B) The percentage of healing measured in 8a′ treated condition. C) Microscopy images of the SNB19 cells and D) its wound healing percentage in SNB19 cells. Data was shown as mean ± S.D (*n* = 5). * indicates statistically significant differences between derivative-treated samples and the DMSO, * *p* < 0.05, ** *p* < 0.001.

To assess the impact of top-lead compound 8a′ on GBM cell migration, the scratch assay was performed using LN229 and SNB19 cell lines. The assay involved creating a linear scratch in cell monolayers, with changes in wound size subsequently monitored every two hours for up to ten hours following treatment with 8a′ at IC_50_ concentration. In the LN229 cell line, treatment with 8a′ did not result in significant wound closure; instead, it led to an increase in wound size by 13% after two hours and 30% after four hours. However, LN229 cells were observed to be in suspension after six hours of treatment (Fig. 3A and B), suggesting a direct cytotoxic effect leading to cellular rounding and detachment. Similarly, an increase in wound size was observed by 8a′ in SNB19 cell line, ranging from 7% to 50% after six hours, followed by complete detachment after eight hours of treatment (Fig. 3C and D). DMSO-treated cells exhibited negligible spontaneous wound closure across all time points. Overall, these data suggest that cell death significantly affected the cell migration.

### 8a′ mediated cell death through EGFR interaction

**Fig. 4:**
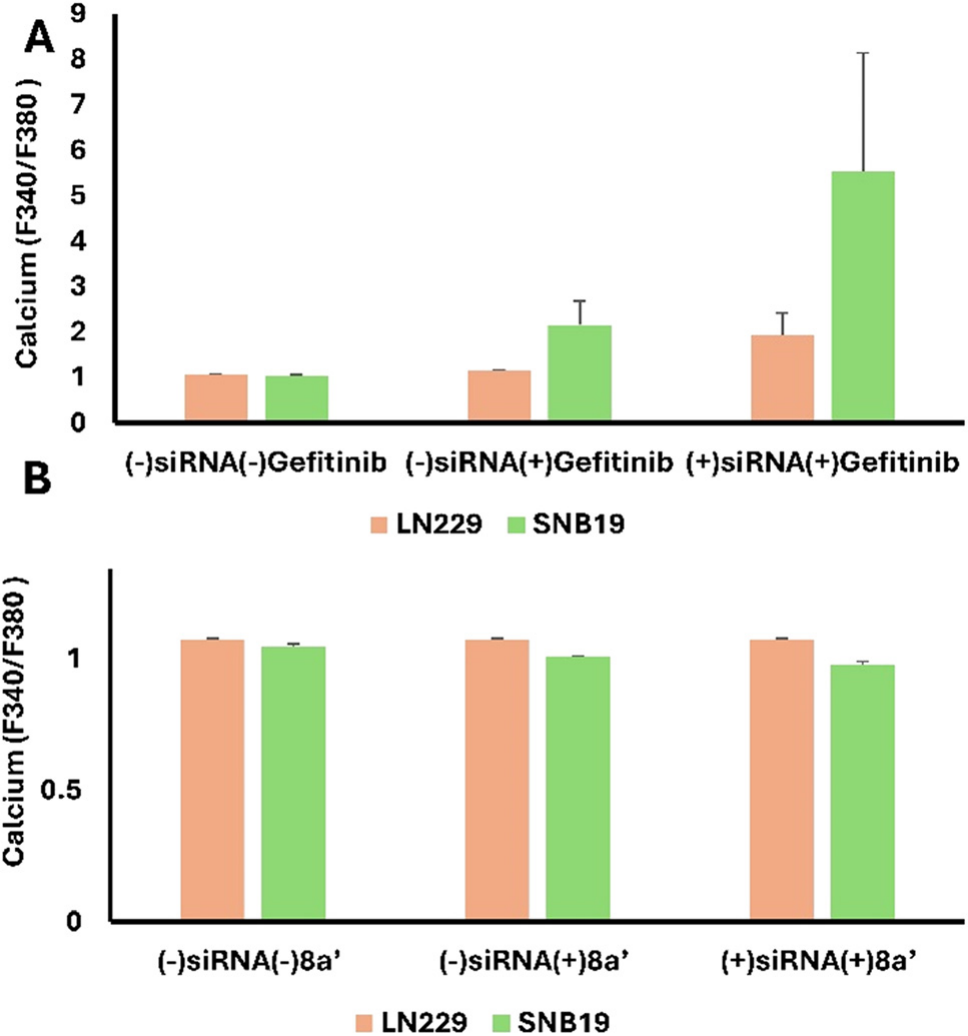
8a′-EGFR regulates intracellular calcium: LN229 and SNB19 cells were treated with 10 μM concentration of gefitinib (positive control) (A) and 8a′ (B) in the presence and absence of siRNA. DMSO was used as a negative control. Calcium level was assessed by Fura 2M assay at the excitation and emission wavelength of *F*_340_/*F*_380_; all data were expressed as the standard error of the mean ± SEM of three independent experiments.

Calcium is a vital secondary messenger, yet excessive intracellular calcium causes cellular malfunction and apoptosis. Increasing evidence reveals that EGFR inhibitors cause intracellular calcium excess and cancer cell death. To determine 8a′-EGFR's Ca^2+^ regulation, LN229 and SNB19 cells were treated with 10 μM doses of 8a′ and EGFR inhibitor gefitinib. Fig. 4A and B illustrate reduced baseline calcium concentration in both LN229 and SNB19 cells in the absence of siRNA with gefitinib or 8a′ treatment, respectively. In Fig. 4A, gefitinib alone significantly increases the calcium level in SNB19 cells compared to LN229 cells. The treatment of gefitinib in the presence of siRNA further enhances calcium levels in SNB19 cells, indicating that EGFR inhibition may activate compensatory pathways such as alternative receptor tyrosine kinases or calcium influx mechanisms that bypass direct EGFR blockades. In Fig. 4B, exposure to 8a′ alone does not significantly alter calcium levels in LN229 cells, but induces a modest reduction in SNB19 cells, indicating cell line-specific sensitivity to this compound. In contrast, treatment with 8a′ in the presence of siRNA further decreases calcium levels in SNB19 cells, whereas LN229 cells remain largely unchanged. Thus, 8a′ might suppress both the EGFR and compensatory signaling mechanisms, leading to a reduction in calcium level. However, LN229 cells maintain relatively stable calcium levels throughout the treatment, implying resistance and varied responses to maintain calcium homeostasis. The above-mentioned findings reveal the cell line-specific differences in EFGR regulation and calcium signaling dynamics, with SNB19 cells exhibiting higher sensitivity, leading to disruption of calcium that drives the induction of apoptosis.

## Conclusions

Notwithstanding the relevance of the Petasis reaction in the preparation of phenolic Mannich bases, the herein-reported formation of triarylmethanes highlights the importance of carefully tuning the reaction conditions. Nevertheless, the synthesis of unsymmetrical triarylmethane remains a topic of research in organic synthesis due to their importance not only in medicinal chemistry but also in the dye industry and materials science. The presence of a halide at the 4-position of the phenolic ring was found to significantly enhance the cytotoxicity of the triarylmethanes under study. Despite their structural similarity, the regioisomer with substitution at the 8-position of the tetrahydroquinoline ring exhibited substantially greater cytotoxicity than its counterpart substituted at the 6-position. Single-crystal X-ray diffraction analysis of the most active compounds provided conclusive evidence confirming the structures of the synthesized phenol derivatives. Derivative 8a′ showed a slight improvement compared to the previously reported 8f′, while being one order of magnitude more potent than standard TMZ. Moreover, the derivative 8a′ presents a double effect on inhibiting cell migration and loss of cell adhesion. The siRNA experiments demonstrate that the unique interaction between 8a′ and EGFR plays a critical role in regulating intracellular calcium in GBM. Work is undergoing to determine the mechanism of action, by transcriptomic analysis, with special emphasis on cell adhesion elements. Additionally, the pharmacological properties of these new compounds will be explored.

## Materials and methods

### Synthesis of triarylmethanes

#### General procedure

The desired compounds were prepared using the multicomponent Petasis borono-Mannich reaction in metal and acid-free conditions. For this, the salicylaldehyde derivative (0.5 mmol) and different substituted aryl boronic acids (0.575 mmol; 1.2 eq.) were dissolved in anhydrous toluene under magnetic stirring in a silicone bath, previously heated at the reaction temperature. 1,2,3,4-Tetrahydroquinoline (125 μL, 1 mmol, 2 eq.) was added using a micro syringe, and the sealed tube was immersed in silicone. The mixture was stirred and refluxed until the complete consumption of the aldehyde, as verified by TLC. The reaction was stopped by cooling to room temperature then the solvent was removed under reduced pressure. The product was purified by chromatography, and the solvent removed under reduced pressure.

#### 8a

Reaction carried out for 3 hours with 5a (78 mg, 0.5 mmol), 6 (109 mg, 0.2 mmol) and 1,2,3,4-tetrahydroquinoline (125 μL, 1 mmol) in toluene (5 mL) at 110 °C. Compound 8a′ was obtained in 23% (47 mg) yield as yellow solid and compound 8a″ in 3% yield (6 mg), upon flash chromatography column using gradient toluene : ethyl acetate (100% to 9 : 1). 8a′: (yellow solid) mp: 155.8–158.7 °C, ^1^H NMR (300 MHz, CDCl_3_): *δ* (ppm) 7.58 (d, *J* = 8.1 Hz, 2H), 7.24 (d, *J* = 8.1 Hz, 2H), 7.11 (dd, *J* = 8.6, 2.6 Hz, 1H), 7.09–6.97 (dd, *J* = 7.4, 1.6 Hz, 1H), 6.80 (d, *J* = 2.6 Hz, 1H), 6.71 (d, *J* = 8.66 Hz, 1H), 6.66 (t, *J* = 7.5 Hz, 1H), 6.56 (dd, *J* = 7.5, 1.7 Hz, 1H), 5.60 (s, 1H), 3.19 (dd, *J* = 6.0, 4.8 Hz, 2H), 2.81 (t, *J* = 6.4 Hz, 2H), 1.86 (dt, *J* = 11.1, 6.4, 4.6 Hz, 2H); ^13^C NMR (75 MHz, CDCl_3_): *δ* (ppm) 152.7, 145.3, 141.5, 130.2, 129.7, 129.6, 129.3 (q, *J*^2^_C–F_ = 33.0 Hz), 129.0, 128.4, 127.4, 125.8, 125.7 (q, *J*^3^_C–F_ = 3.3 Hz), 125.6, 124.2 (q, *J*_C–F_ = 270.0 Hz), 123.7, 118.0, 117.7, 45.6, 42.5, 27.5, 21.8; ^19^F NMR (282 MHz, CDCl_3_): *δ* (ppm) −65.59; HRMS *m*/*z*: [M + H]^+^ calculated for C_23_H_20_ClF_3_NO^+^, 418.1186; found 418.1174. 8a″ (yellow oil): ^1^H NMR (300 MHz, CDCl_3_): *δ* (ppm) 7.55 (d, *J* = 8.1 Hz, 2H), 7.23 (d, *J* = 8.1 Hz, 2H), 7.10 (dd, *J* = 8.5, 2.6 Hz, 1H), 6.77–6.72 (m, 2H), 6.70–6.63 (m, 2H), 6.43 (dd, *J* = 7.5, 1.4 Hz, 1H), 5.53 (s, 1H), 3.34–3.25 (m, 2H), 2.69 (t, *J* = 6.4 Hz, 2H), 1.92 (dt, *J* = 11.1, 6.4, 5.8 Hz, 2H); ^13^C NMR (75 MHz, CDCl_3_): *δ* (ppm) 152.2, 147.0, 147.0, 143.8, 132.2, 130.3, 130.0, 129.6, 129.1, 128.7 (q, *J*^2^_C–F_ = 32.0 Hz), 127.8, 127.5, 125.6, 125.4 (q, *J*^3^_C–F_ = 3.7 Hz), 124.3 (q, *J*_C–F_ = 270.0 Hz), 122.2, 117.4, 114.8, 50.0, 42.0, 26.9, 21.9; ^19^F NMR (282 MHz, CDCl_3_): *δ* (ppm) −65.55.

#### 8b′ (yellow solid)

Reaction carried out for 7 hours, using 5b (101 mg, 0.5 mmol), 6 (109 mg, 0.6 mmol) and 1,2,3,4-tetrahydroquinoline (125 μL, 1 mmol) in toluene (5 mL) at 135 °C. The compound was obtained as a yellow solid in 32% yield (74 mg) after purification by flash chromatography column using a gradient toluene : ethyl acetate (100% to 9 : 1). ^1^H NMR (300 MHz, CDCl_3_): *δ* (ppm) 7.57 (d, *J* = 8.1 Hz, 2H), 7.29–7.21 (m, 3H), 6.99–6.90 (m, 2H), 6.71 (d, *J* = 8.5 Hz, 1H), 6.64 (t, *J* = 7.5 Hz, 1H), 6.55 (dd, *J* = 7.5, 1.7 Hz, 1H), 5.56 (s, 1H), 3.21 (dd, *J* = 6.0, 4.8 Hz, 2H), 2.80 (t, *J* = 6.4 Hz, 2H), 1.86 (dt, *J* = 11.2, 6.4, 4.6 Hz, 2H); ^13^C NMR (75 MHz, CDCl_3_): *δ* (ppm) 153.3, 145.4, 141.7, 132.7, 131.4, 130.9, 129.6, 129.3 (q, *J*^2^_C–F_ = 32.0 Hz), 129.1, 127.6, 126.1, 125.8 (q, *J*^3^_C–F_ = 3.8 Hz), 124.3 (q, *J*_C–F_ = 270.0 Hz), 123.8, 118.3, 118.1, 113.2, 45.8, 42.6, 27.6, 21.9; ^19^F NMR (282 MHz, CDCl_3_): *δ* (ppm) −65.59; HRMS *m*/*z*: [M + H]^+^ calculated for C_23_H_20_BrF_3_NO^+^, 462.0680; found 462.0669.

#### 8c′ (light yellow solid)

Reaction was carried out for 2 hours, using 5c (70 mg, 0.5 mmol), 6 (109 mg, 0.6 mmol), and 1,2,3,4-tetrahydroquinoline (125 μL, 1 mmol) in toluene (5 mL) at 110 °C. The compound was obtained as a light yellow solid in 41% yield (82 mg) after purification by flash chromatography column using hexane : ethyl acetate (8 : 2) as eluent. mp: 145.3–147.1 °C, ^1^H NMR (300 MHz, CDCl_3_): *δ* (ppm) 7.57 (d, *J* = 8.2 Hz, 2H), 7.25 (d, *J* = 8.2 Hz, 2H), 6.94 (dd, *J* = 7.4, 1.5 Hz, 1H), 6.87 (ddd, *J* = 8.8, 7.7, 3.0 Hz, 1H), 6.77 (m, 1H), 6.61 (t, *J* = 7.5 Hz, 1H), 6.55–6.48 (m, 2H), 5.56 (s, 1H), 3.22 (dd, *J* = 6.0, 4.8 Hz, 2H), 2.80 (t, *J* = 6.4 Hz, 2H), 1.87 (dt, *J* = 11.2, 6.4, 4.6 Hz, 2H); ^13^C NMR (75 MHz, CDCl_3_): *δ* (ppm) 157.3 (d, *J*_C–F_ = 238.8 Hz), 149.8 (d, *J*^4^_C–F_ = 2.2 Hz), 145.4, 141.9, 130.2 (d, *J*^3^_C–F_ = 6.6 Hz), 129.7, 129.0 (q, *J*^2^_C–F_ = 32.0 Hz), 129.0, 127.2, 125.6 (q, *J*^3^_C–F_ = 3.9 Hz), 125.3 (q, *J*_C–F_ = 270.0 Hz), 125.3, 123.2, 117.5, 117.2 (d, *J*^3^_C–F_ = 8.0 Hz), 116.5 (d, *J*^2^_C–F_ = 24.0 Hz), 114.74 (d, *J*^2^_C–F_ = 23.2 Hz), 45.5, 42.5, 27.5, 21.8; ^19^F NMR (282 MHz, CDCl_3_): *δ* (ppm) −65.59, −125.88 (td, *J* = 8.7, 4.9 Hz); HRMS *m*/*z*: [M + H]^+^ calculated for C_23_H_20_F_4_NO^+^, 402.1481; found 402.1472.

#### 8d′ (yellow solid)

Reaction was carried out for 3 hours, using 8d (68 mg, 0.5 mmol), 6 (109 mg, 0.6 mmol), and 1,2,3,4-tetrahydroquinoline (125 μL, 1 mmol) in toluene (5 mL) at 135 °C. The compound was obtained in 44% yield (87 mg) as a yellow solid after flash column chromatography using dichloromethane : hexane (7 : 3) as eluent. mp: 147.6–152.7 °C, ^1^H NMR (300 MHz, CDCl3): *δ* (ppm) 7.55 (d, *J* = 8.1 Hz, 2H), 7.25 (d, *J* = 8.1 Hz, 2H), 7.00–6.90 (m, 2H), 6.72 (d, *J* = 8.1 Hz, 1H), 6.64–6.56 (m, 2H), 6.51 (dd, *J* = 7.5, 1.3 Hz, 1H), 5.56 (s, 1H), 3.21 (dd, *J* = 6.4, 4.6 Hz, 2H), 2.80 (t, *J* = 6.4 Hz, 2H), 2.19 (s, 3H), 1.86 (dt, *J* = 11.0, 6.4, 4.6 Hz, 2H); ^13^C NMR (75 MHz, CDCl_3_): *δ* (ppm) 151.6, 146.3, 142.1, 130.5, 130.3, 129.8, 129.0, 128.8 (q, *J*^2^_C–F_ = 38.0 Hz), 128.6, 127.8, 127.4, 126.1, 125.5 (q, *J*^3^_C–F_ = 3.8 Hz), 124.3 (q, *J*_C–F_ = 270.0 Hz), 122.9, 117.2, 116.2, 45.7, 42.4, 27.5, 21.8, 20.7; ^19^F NMR (282 MHz, CDCl_3_): *δ* (ppm) −65.52 HRMS *m*/*z*: [M + H]^+^ calculated for C_24_H_23_F_3_NO^+^, 398.1731; found 398.1698.

#### 8e′ (light yellow solid)

Reaction was carried out for 5 hours using 5e (62 μL, 0.5 mmol), 6 (109 mg, 0.6 mmol), and 1,2,3,4-tetrahydroquinoline (125 μL, 1 mmol) in toluene (5 mL) at 135 °C. The compound was obtained in 41% yield (85 mg) after purification by flash chromatography column using a gradient of toluene : ethyl acetate (9 : 1 → 8 : 2). ^1^H NMR (300 MHz, CDCl_3_): *δ* (ppm) 7.55 (d, *J* = 8.2 Hz, 2H), 7.26 (d, *J* = 8.2 Hz, 2H), 6.92 (dd, *J* = 7.5, 1.6 Hz, 1H), 6.81–6.67 (m, 2H), 6.58 (t, *J* = 7.5 Hz, 1H), 6.51 (dd, *J* = 7.5, 1.3 Hz, 1H), 6.38 (d, *J* = 2.9 Hz, 1H), 5.56 (s, 1H), 3.67 (s, 3H), 3.21 (dd, *J* = 6.4, 4.6 Hz, 2H), 2.79 (t, *J* = 6.4 Hz, 2H), 1.86 (dt, *J* = 11.2, 6.4, 5.6 Hz, 2H); ^13^C NMR (75 MHz, CDCl_3_): *δ* (ppm) 153.8, 147.8, 145.9, 142.0, 129.8, 129.6, 129.0 (q, *J*^2^_C–F_ = 32.0 Hz), 128.8, 127.3, 125.7, 125.5 (q, *J*^3^_C–F_ = 3.9 Hz), 124.2 (q, *J*_C–F_ = 270.0 Hz), 122.9, 117.3, 117.0, 116.3, 112.5, 55.6, 45.7, 42.5, 27.5, 21.8; ^19^F NMR (282 MHz, CDCl_3_): *δ* (ppm) −65.56; HRMS *m*/*z*: [M + H]^+^ calculated for C_24_H_23_F_3_NO_2_^+^, 414.1681; found 414.1646.

#### 8f′ (yellow solid)

Reaction was carried out as previously described,^22^ for 3 hours using 5f (52 μL, 0.5 mmol), 6 (109 mg, 0.6 mmol), and 1,2,3,4-tetrahydroquinoline (125 μL, 1 mmol) in toluene (5 mL) at 135 °C. The compound was obtained in 61% yield (117 mg) after purification by flash chromatography column using a gradient of hexane : ethyl acetate. mp: 127.5–132.5 °C, ^1^H NMR (300 MHz, CDCl_3_): *δ* (ppm) 7.44 (d, *J* = 8.0 Hz, 2H), 7.13 (d, *J* = 8.0 Hz, 2H), 7.09–7.01 (m, 1H), 6.84 (dd, *J* = 7.4, 1.5 Hz, 1H) 6.81–6.63 (m, 4H), 6.51 (t, *J* = 7.5 Hz, 1H), 6.42 (dd, *J* = 7.7, 1.5 Hz, 1H), 5.55 (s, 1H), 3.18–3.01 (m, 2H), 2.69 (t, *J* = 6.4 Hz, 2H), 1.85–1.67 (m, 2H). ^13^C NMR (CDCl_3_, 75 MHz): *δ* (ppm) 153.9, 146.41, 146.39, 141.8, 130.1, 129.8–118.9 (q, *J*^2^_C–F_ = 270 Hz), 129.5–128.2 (q, *J*^3^_C–F_ = 38 Hz), 128.5, 127.5, 126.8, 125.5–125.4 (q, *J*^3^_C–F_ = 45 Hz), 123.2, 120.9, 117.6, 116.2, 45.4, 42.5, 27.5, 21.9. ^19^F NMR (282 MHz, CDCl_3_): *δ* (ppm) −65.54; HRMS (ESI^+^) calculated for C_23_H_21_F_3_NO [M + H]^+^ 384.1575; found 384.1542.

#### 9 (yellow solid)

Benzoyl chloride (16 μL, 0.14 mol) was added dropwise to a 0 °C cooled solution of 8a″ (30 mg, 0.07 mmol) and triethylamine (20 μL, 0.14 mmol) in anhydrous dichloromethane (1 mL). After 1 hour at such temperature, the reaction mixture was allowed to reach room temperature and left stirring for 3 h. After solvent removal, the mixture was purified by column chromatography using hexane : ethyl acetate (gradient: 95 : 5 to 90 : 10). ^1^H NMR (300 MHz, CDCl_3_): *δ* (ppm) 7.87–7.79 (m, 2H), 7.61 (tt, *J* = 7.5, 1.2 Hz, 1H), 7.48 (d, *J* = 8.4 Hz, 2H), 7.45–7.28 (m, 8H), 7.18–7.08 (m, 3H), 6.82 (d, *J* = 2.7 Hz, 1H), 6.79 (d, *J* = 2.1 Hz, 1H), 6.71 (d, *J* = 8.6 Hz, 1H), 6.55 (dd, *J* = 8.4, 2.2 Hz, 1H), 5.54 (s, 1H), 3.87 (t, *J* = 6.4 Hz, 2H), 2.82–2.59 (m, 2H), 2.05–1.90 (m, 2H); ^13^C NMR (75 MHz, CDCl_3_): *δ* (ppm) 170.44, 164.32, 147.51, 138.41, 137.37, 136.81, 136.27, 134.02, 131.81, 131.67, 130.45, 130.38, 130.14, 129.60, 129.32, 128.69, 128.65, 128.45 (q, *J* = 32.0 Hz), 128.34, 128.27, 126.72, 125.57 (q, *J* = 3.3 Hz), 124.54, 124.36 (q, *J* = 270.0 Hz), 50.75, 27.10, 24.08. ^19^F NMR (282 MHz, CDCl_3_): *δ* (ppm) −61.65.

### Cell culture

Human glioblastoma (GBM) cell lines, LN229 and SNB19 (gifted by Prof. Maria Stella Carro, University Medical Center Freiburg, Germany), were used to investigate the effect of tetrahydroquinoline-derived triarylmethanes. The LN229 line, established in 1979 from the right frontal parieto-occipital cortex of a glioblastoma patient, harbors a TP53 mutation and homozygous deletions in p16 and p14ARF tumor suppressor genes. The SNB19 line, established in 1980 from a left parieto-occipital glioblastoma, presents with mutations in both the PTEN and TP53 genes. Both cell lines were cultured in Dulbecco's modified Eagle medium (DMEM) supplemented with 10% fetal bovine serum (FBS), 0.1 mg mL^−1^ streptomycin, and 100 U mL^−1^ penicillin (all reagents from Sigma-Aldrich, St. Louis, MO, United States). Cells were maintained at 37 °C in a humidified atmosphere with 5% CO_2_.

### Cytotoxicity assay

The cytotoxicity of seven newly synthesized triarylmethane derivatives against GBM cell proliferation was assessed using the Trypan blue exclusion assay. LN229 cells were seeded in a 12 well-plate with the density of 1 × 10^5^ cells per well. At 60–70% confluency, cells were treated with each derivative at a concentration of 50 μM and incubated for 48 hours in a CO_2_ incubator. The at 50 μM of temozolomide (TMZ), a frontline chemotherapeutic drug for malignant glioma, was used as positive control, and 0.1% DMSO served as a negative control. Cell growth inhibition (%) was quantified using the Countess II FL Automated Cell Counter (ThermoFisher Scientific, Carlsbad, CA, USA). This percentage was calculated using the following formula:
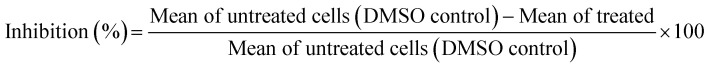


### Inhibitory kinetic study

An inhibitory kinetic study was conducted to evaluate the dose-dependent cell growth inhibition of the top compounds and TMZ on LN229 and SNB19 cell lines over a 48 h exposure period. GBM cells were treated with concentrations of 1, 10, 25, 50, 75, 100 μM and a 0.1% DMSO solution served as a negative control. Following treatment, the percentage of cell growth inhibition was determined using the Trypan blue exclusion assay, as described above. Dose–response curves were plotted to determine the half maximal inhibitory concentration (IC_50_) for each cell line. Additionally, a time-dependent cytotoxicity analysis was conducted using the IC_50_ concentrations specific to each cell line at 24 and 48 hours, following the previously described protocol.

### Wound healing assay

To evaluate the cell migration activity of top compound, confluent monolayers of cells in 12-well plates were prepared, seeding approximately 3 × 10^5^ cells per well. Following the scratch made in each well, any detached or floating cells were removed by washing with PBS. The cells were then treated with either the IC_50_ concentration of the top compound or with DMSO as a negative control. The scratch area was captured every 2 h for a period of 10 h using a EVOS Cell Imaging System and the images were then analyzed with ImageJ software.

### Calcium kinetic assay

To perform calcium assay, 1 × 10^4^ density of LN229 and SNB19 cells per well were seeded into a black, clear-bottom, 96-well plate. To validate the specificity of the ligand binding to the EGFR receptor, a gene silencing approach utilizing small interfering RNA (siRNA) was implemented. Pre-designed siRNA targeting human EGFR was commercially synthesized and acquired from (Thermo Fisher Scientific, USA). Both GBM cell lines, were seeded with the confluence of 60–70% and transfected with 20 nM of siRNA using Lipofectamine RNAiMAX Transfection Reagent (Thermo Fisher Scientific, USA) according to the manufacturer's protocol. Following a 48-hour incubation, the calcium levels were measured to quantify the alterations in intracellular calcium concentration in response to the experimental compound. The growth medium was replaced with 100 μL of Fura-2 AM and 0.01% Pluronic® F-127 (Abcam) in Hanks' buffer with supplemented with HEPES (HHBS). Cells were incubated at 37 °C for 1 hour, followed by an additional 20 minutes at room temperature. Subsequently, 10 μM final concentration of 8a′ and gefitinib were prepared in HHBS and added directly to the wells. Fluorescence measurements were acquired using a microplate reader (SPARK®, TECAN, Mannedorf, Switzerland) at 37 °C. Excitation was performed at 340 nm and 380 nm, and emission was recorded at 510 nm. The changes in intracellular calcium concentration were calculated using the following formula:
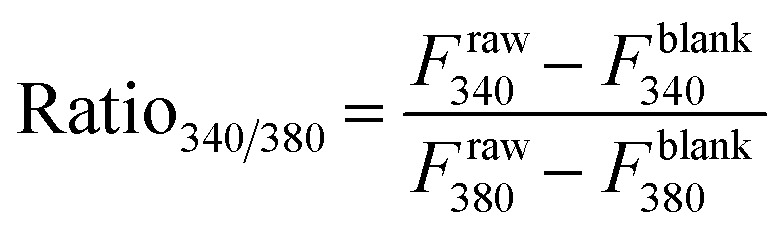
The intracellular calcium in this assay was quantified by the value *F*_340_/*F*_380_, which directly represents the fold change of it. This ratio is calculated using the emitted fluorescence intensities of the sample collected at the two excitation wavelengths: *F*^raw^_340_ (measured at 340/510 nm) and *F*^raw^_380_ (measured at 380/510 nm). To ensure accurate signal measurement, both raw intensities were corrected using background values (*F*^blank^_340_ and *F*^blank^_380_).

## Author contributions

DSNB and ZHY: formal analysis, investigation and methodology; FAAP and SC: data curation and visualization; MK and NRC: conceptualization, supervision, writing (original draft, review & editing).

## Conflicts of interest

There are no conflicts to declare.

## Supplementary Material

MD-OLF-D5MD00585J-s001

MD-OLF-D5MD00585J-s002

## Data Availability

The data supporting this article have been included as part of the supplementary information (SI). Supplementary information: additional experiments, ^1^H and ^13^C NMR, and X-ray data. See DOI: https://doi.org/10.1039/d5md00585j. CCDC 2468967–2468969 (8a′, 9 and 8f′) contain the supplementary crystallographic data for this paper.^44*a*–*c*^
