# Supplementary material for: Glioblastoma antitumoral activity of tetrahydroquinoline-derived triarylmethanes
Source: RSC Med Chem. 2025 Oct 24;16(12):6204–13. doi: 10.1039/d5md00585j (PMC12613095; doi:10.1039/d5md00585j)
Supplement: MD-016-D5MD00585J-s001 [file MD-016-D5MD00585J-s001.pdf]

Supplementary Information (SI) for RSC Medicinal Chemistry.
This journal is © The Royal Society of Chemistry 2025

Glioblastoma Antitumoral Activity of Tetrahydroquinoline-derived Triarylmethanes

Daniela S. N. Branco[a], Zahra Hosseinpur Yektaei[b,c], Sureka Chandrabose[d], Filipe A. Almeida Paz,[e] Meenakshisundaram Kandhavelu*[b,c] and Nuno R. Candeias *[a,f]

Additional screening experiments 2
Single-Crystal X-Ray Diffraction Studies 3
NMR spectra 5

Additional screening experiments

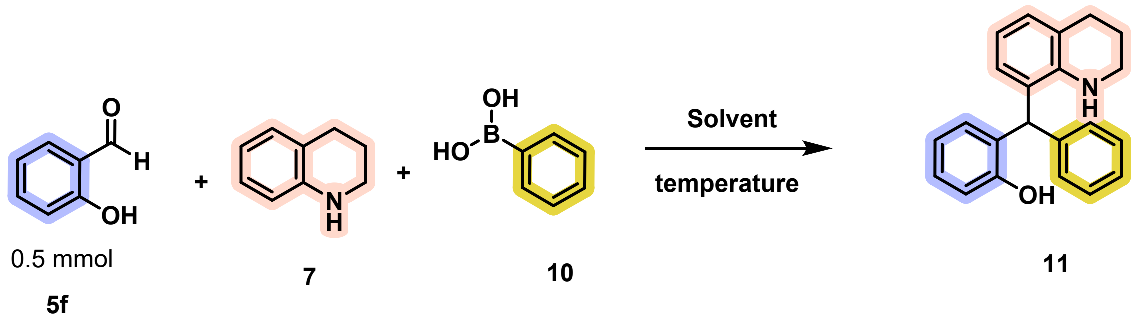

Entry	Stoichiometry 5f : 7 : 10	Solvent	[5f] (M)	Temperature (ºC)	Reaction time (h)	Isolated Yield (%)
1	1 : 2 : 1.2	Toluene	0.1 M	135	1	62
2	1 : 2 : 1.2	$C_2H_4Cl_2$	0.3 M	100	23	63
3	1 : 2 : 1.2	EtOH	0.1 M	135	23	-
4	1 : 2 : 1.2	CH_3CN	0.3 M	100	5	20
5	1 : 1.2 : 1	Toluene	0.3 M	135	22	53
6	2 : 1 : 1.2	Toluene	0.7 M	135	21	35
7	1 : 1.5 : 2	CH_2Cl_2[a]	0.3 M	rt	24	43
8	2 : 1 : 1.2	CH_2Cl_2[a]	0.5 M	rt	46	33

[a] in presence of 160 mg of molecular sieves

Table S1 – Screening of conditions for formation of tetrahydroquinoline-derived TAM.

Single-Crystal X-Ray Diffraction Studies

Single crystals of compounds **8a'**, **9** and **8f'** were manually harvested from the crystallization vials and immersed in highly viscous FOMBLIN Y perfluoropolyether vacuum oil (LVAC 140/13, Sigma-Aldrich) to avoid degradation caused by the evaporation of the solvent.[1] Crystals were mounted on either Hampton Research CryoLoops or MiTeGen MicroLoops, typically with the help of a Stemi 2000 stereomicroscope equipped with Carl Zeiss lenses.

X-ray diffraction data for all compounds were recorded at 150(2) K with an XtaLAB Synergy-i (Rigaku, Tokyo, Japan) diffractometer equipped with a Cryostream 800 (Oxford Cryosystems) and a HiPix3000 Bantam detector. Data were collected using a monochromatized microfocus PhotonJet-I MoKα radiation source (λ = 0.71073 Å). Evaluation, integration, and reduction of the diffraction data were carried out with the CrysAlis Pro software suite.[2]

All structures were solved using the algorithm implemented in SHELXT-2014/5,[3] which allowed the immediate location of almost all of the heaviest atoms composing the molecular unit of the four compounds. The remaining missing and misplaced non-hydrogen atoms were located from difference Fourier maps calculated from successive full-matrix least-squares refinement cycles on F^2 using the latest SHELXL from the 2018/3 release.[4] All structural refinements were performed using the graphical interface ShelXle.[5]

Hydrogen atoms bound to carbon and oxygen were placed at their idealized positions using appropriate *HFIX* instructions in SHELXL: *43* (aromatic carbon atoms), *13* (tertiary carbon atoms), *23* (–CH_2– carbon atoms), *147* (for the hydroxyl groups). These hydrogen atoms were included in subsequent refinement cycles with isotropic thermal displacements parameters (U_{iso}) fixed at 1.2 or 1.5×U_{eq} (only for the later family of groups) of the parent non-hydrogen atoms.

For compound **8f'** the terminal C–F_3 moiety was found to be severely affected by positional disorder. The group was included in the final structural model with the F-atoms distributed among three distinct positions whose rates of occupancy were fixed (at later stages of the refinement) as 30%, 40% and 30% (please note: rates determined from unrestrained refinement cycles). These atoms could not be modelled using anisotropic displacement parameters being instead included in the structural model using independent isotropic displacement parameters.

The last difference Fourier map synthesis showed: for **8a'**, the highest peak (0.444 eÅ^{-3}) and the deepest hole (-0.506 eÅ^{-3}) located at 0.86 and 0.75 Å from Cl1, respectively; for **9**, the highest peak (0.361 eÅ^{-3}) and the deepest hole (-0.303 eÅ^{-3}) located at 1.18 and 0.82 Å from F3, respectively; for **8f'**, the highest peak (0.769 eÅ^{-3}) and the deepest hole (-0.472 eÅ^{-3}) located at 0.79 and 0.58 Å from F7 and F5, respectively.

Structural drawings have been created using the software package Crystal Impact Diamond.[6]

Crystal data for **8a'**: $C_{23}H_{19}ClF_3NO$, M = 417.84, monoclinic, space group $P2_1$/c, Z = 4, a = 11.45488(17) Å, b = 17.2029(3) Å, c = 9.67733(13) Å, β = 97.5738(13)º, V = 1890.35(5) Å^3, μ(Mo-Kα) = 0.246 mm^{-1}, D_c = 1.468 g cm^{-3}, yellow block with crystal size of 0.20×0.18×0.13 mm^3. Of a total of 22476 reflections collected, 3385 were

independent (R_{int} = 0.0199). Final $R1$ = 0.0333 [$I > 2\sigma(I)$] and $wR2$ = 0.0851 (all data). Data completeness to theta = 25.24°, 98.2%. CCDC 2468967.

Crystal data for **9**: $C_{37}H_{27}ClF_3NO_3$, M = 626.04, triclinic, space group P-1, Z = 2, a = 7.9684(2) Å, b = 12.2691(3) Å, c = 16.0679(4) Å, α = 78.803(2)°, β = 79.663(2)°, γ = 76.349(2)°, V = 1482.67(7) Å^3, μ(Mo-Kα) = 0.188 mm^{-1}, D_c = 1.402 g cm^{-3}, yellow block with crystal size of 0.08×0.07×0.02 mm^3. Of a total of 31555 reflections collected, 5416 were independent (R_{int} = 0.0254). Final $R1$ = 0.0393 [$I > 2\sigma(I)$] and $wR2$ = 0.1008 (all data). Data completeness to theta = 25.24°, 99.7%. CCDC 2468969.

Crystal data for **8f'**: $C_{23}H_{20}F_3NO$, M = 383.40, monoclinic, space group $P2_1$/c, Z = 4, a = 11.1723(2) Å, b = 16.3213(4) Å, c = 10.3436(2) Å, β = 98.802(2)°, V = 1863.91(7) Å^3, μ(Mo-Kα) = 0.104 mm^{-1}, D_c = 1.366 g cm^{-3}, yellow plate with crystal size of 0.20×0.16×0.10 mm^3. Of a total of 30525 reflections collected, 4445 were independent (R_{int} = 0.0234). Final $R1$ = 0.0663 [$I > 2\sigma(I)$] and $wR2$ = 0.2031 (all data). Data completeness to theta = 25.24°, 100.0%. CCDC 2468968.

References

1. T. Kottke and D. Stalke, *J. Appl. Crystallogr.*, 1993, **26**, 615-619.
2. CrysAlisPro, *Version 1.171.43.143a, Rigaku Oxford Diffraction, Oxford, UK,*, 2024.
3. G. M. Sheldrick, *Acta Cryst. A*, 2015, **71**, 3-8.
4. G. M. Sheldrick, *Acta Cryst. C*, 2015, **71**, 3-8.
5. C. B. Hübschle, G. M. Sheldrick and B. Dittric, *J. Appl. Crystallogr.*, 2011, **44**, 1281-1284.
6. K. Brandenburg, *DIAMOND, Version 3.2f. Crystal Impact GbR, Bonn, Germany*, 1997-2010.

NMR spectra

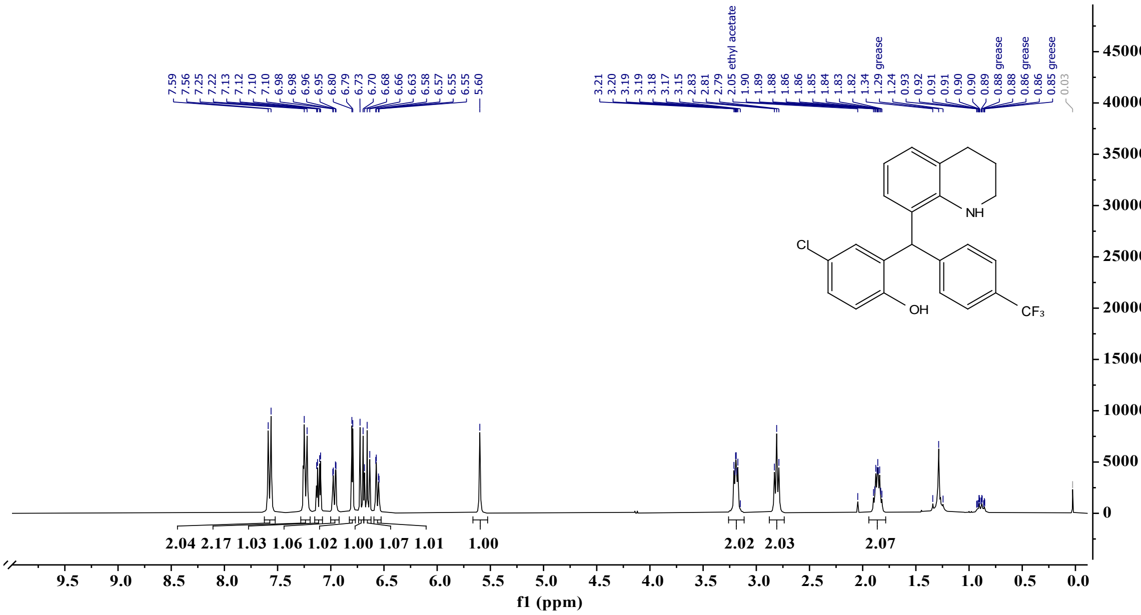

Figure S1: ^{1}H NMR of **8a'** ($CDCl_3$, 300 MHz).

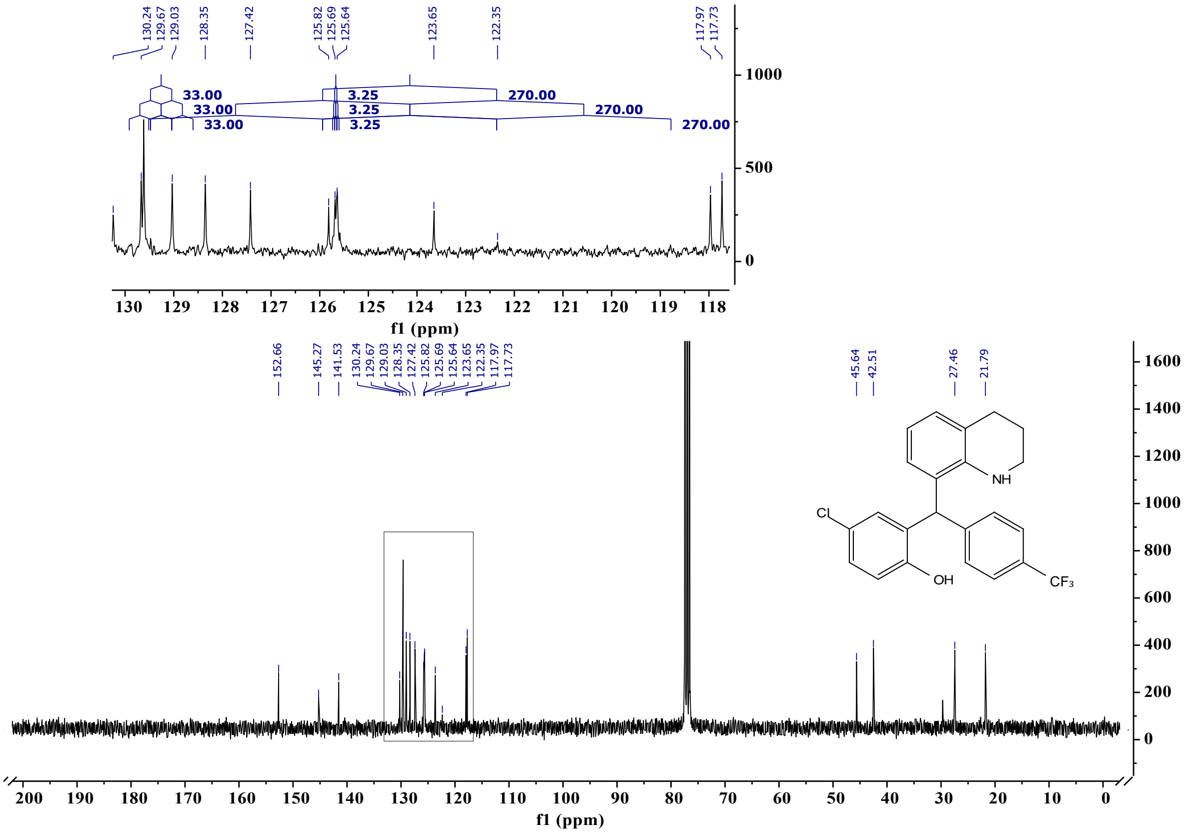

Figure S2:^{13}C NMR of **8a'** ($CDCl_3$, 75 MHz).

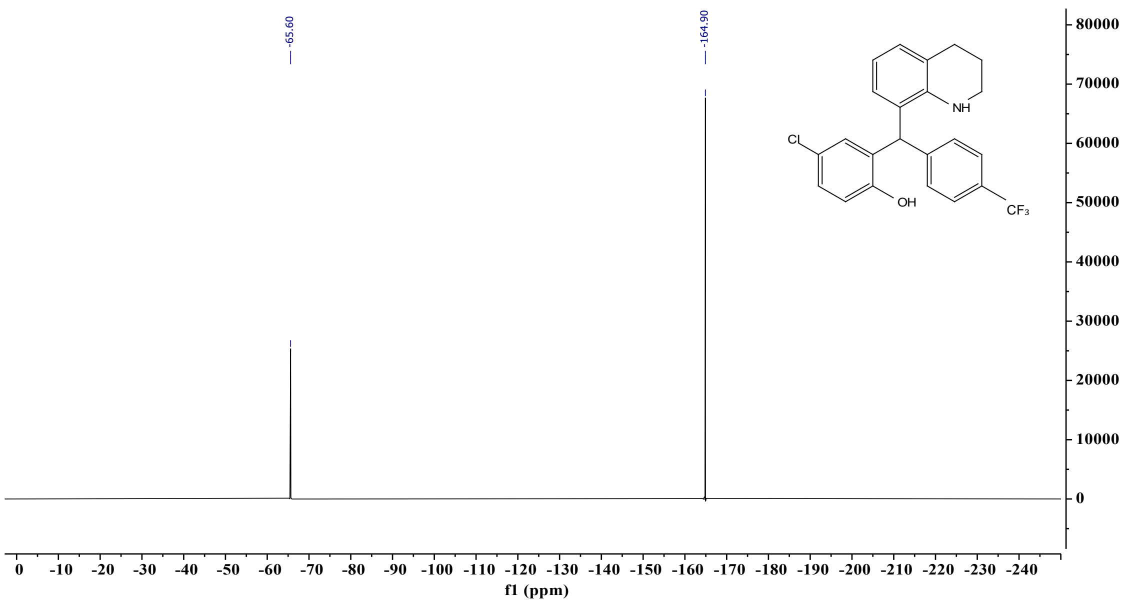

Figure S3: ^{19}F NMR of **8a'** ($CDCl_3$, 282 MHz), with C_6F_6 internal standard (δ -164.9 ppm).

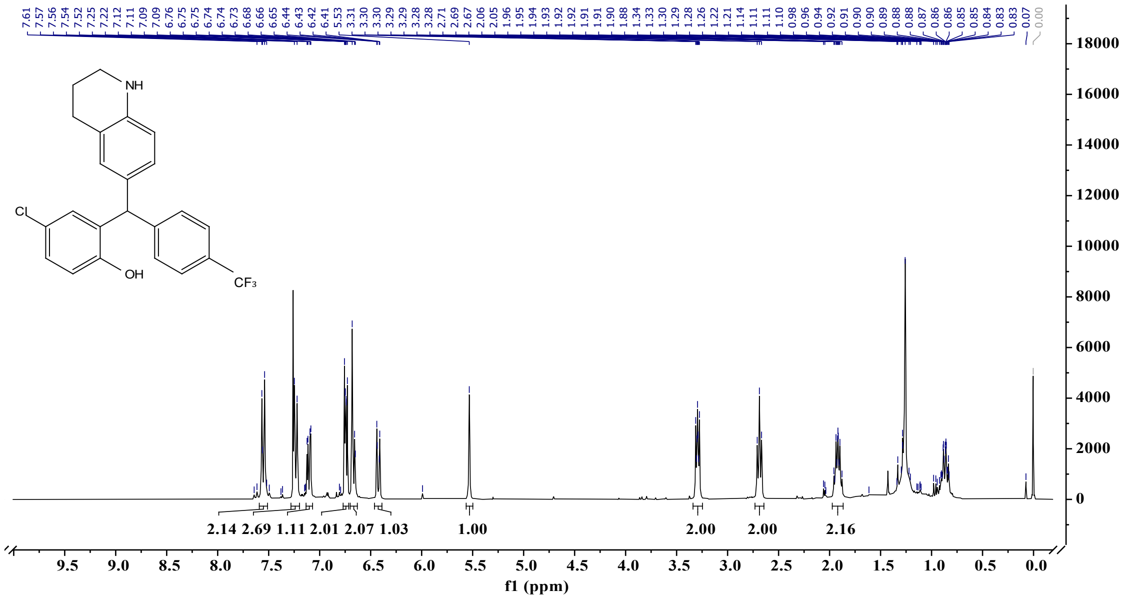

Figure S4:^{1}H NMR of **8a''** ($CDCl_3$, 300 MHz).

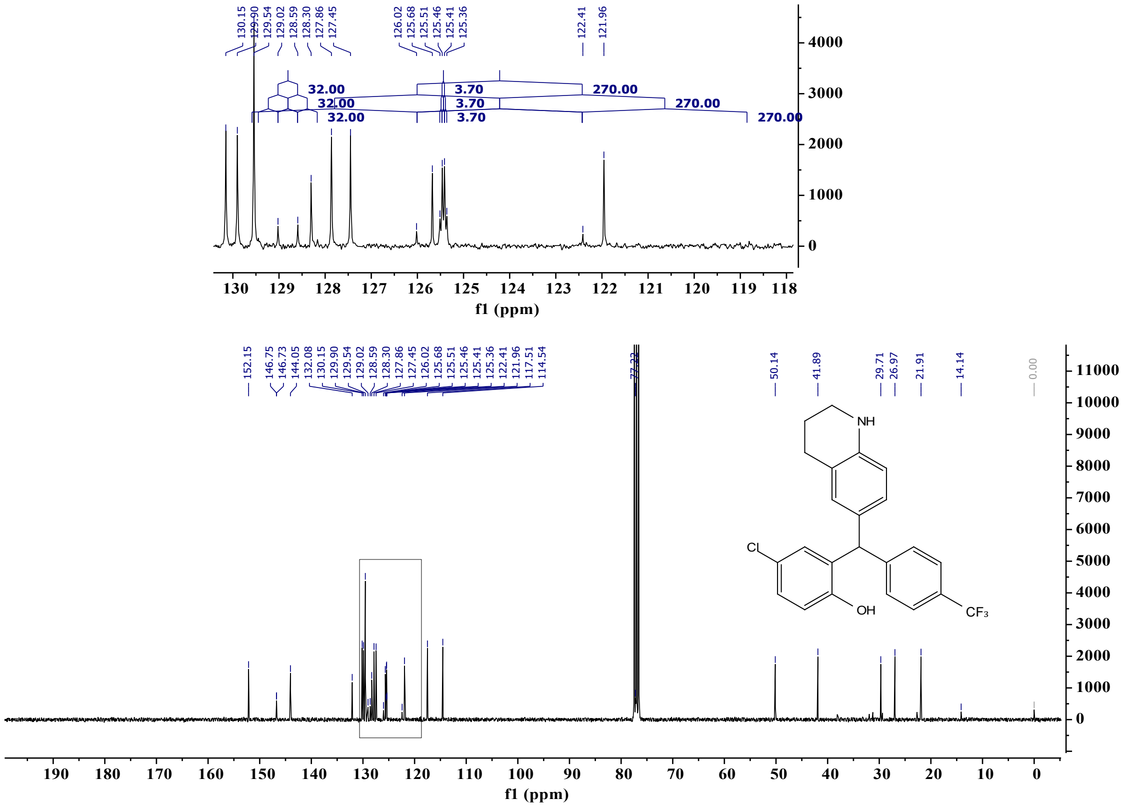

Figure S5:^{13}C NMR of **8a''** ($CDCl_3$, 75 MHz).

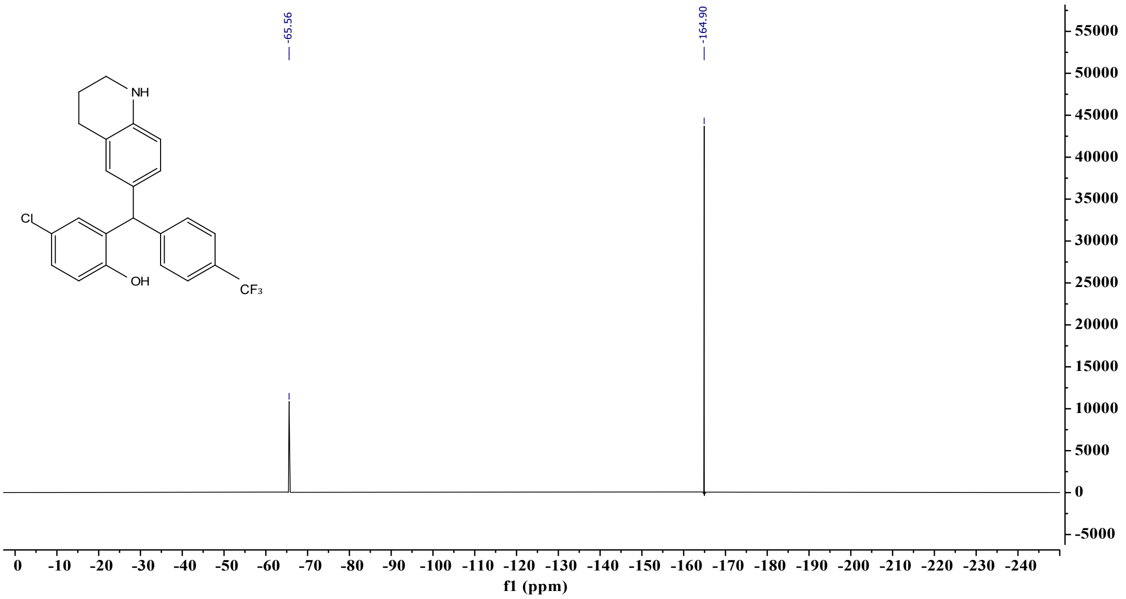

Figure S6:^{19}F NMR of **8a''** ($CDCl_3$, 282 MHz), with C_6F_6 internal standard (δ -164.9 ppm).

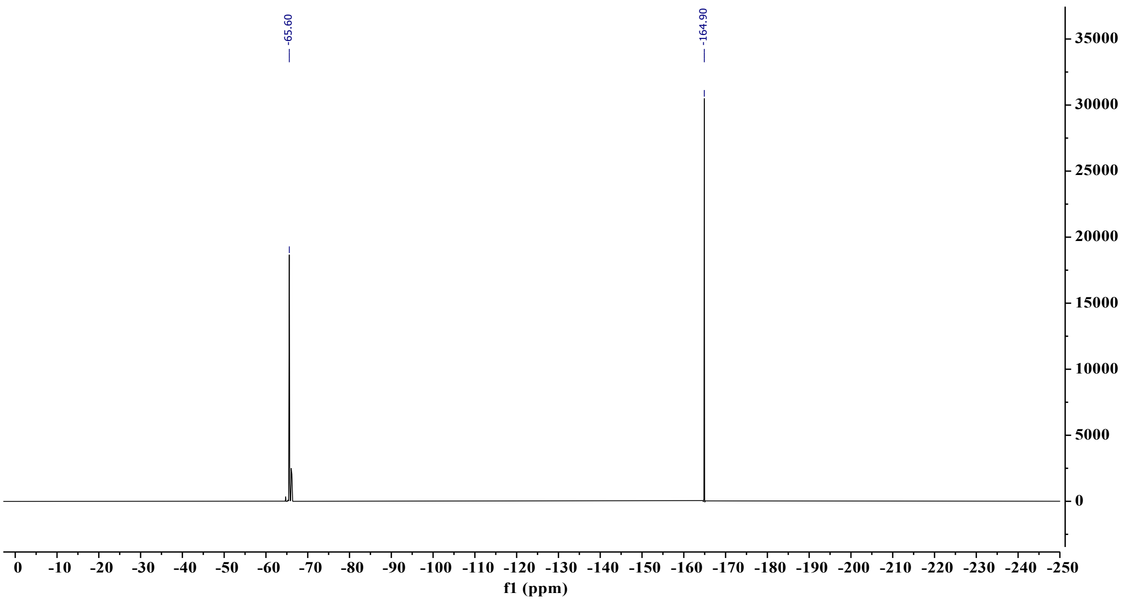

Figure S7:^{19}F NMR crude spectra for reaction with **5a** ($CDCl_3$, 282 MHz), with C_6F_6 internal standard (δ -164.9 ppm).

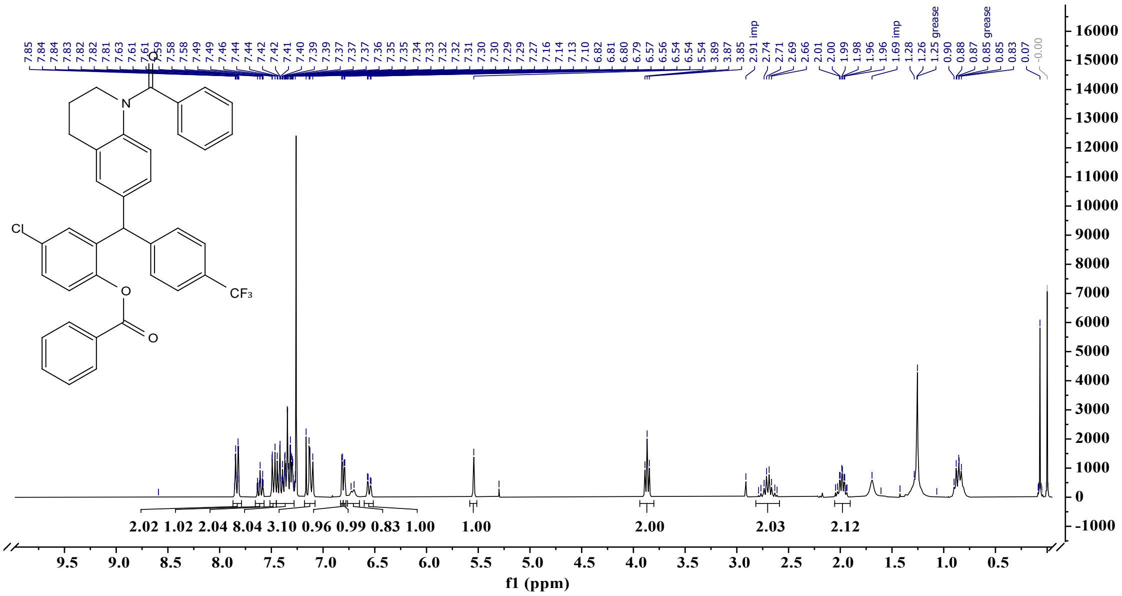

Figure S8:^{1}H NMR of **9** ($CDCl_3$, 300 MHz).

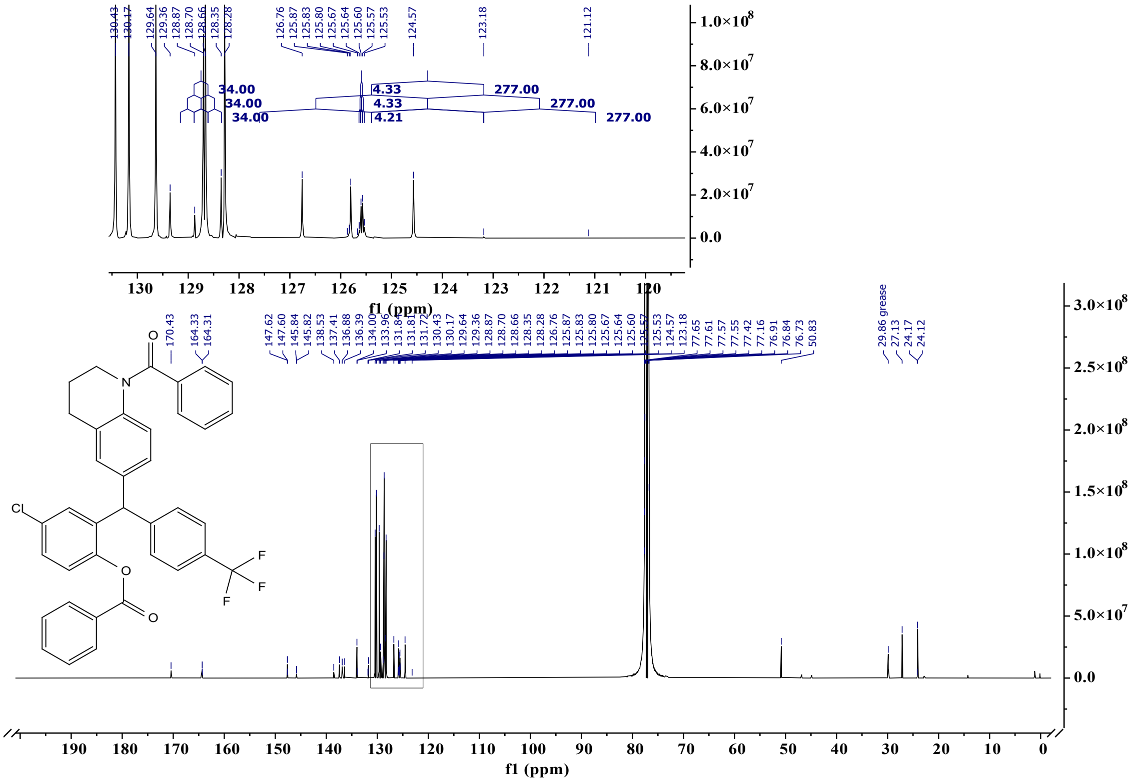

Figure S9:^{13}C NMR of **9** ($CDCl_3$,126 MHz).

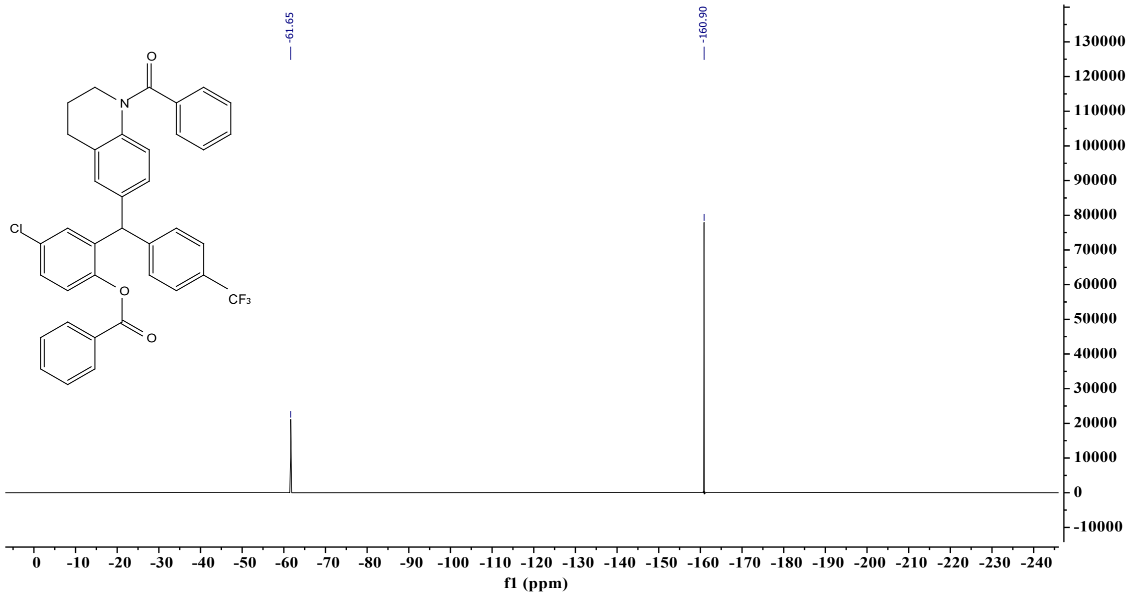

Figure S10:^{19}F NMR of **9** ($CDCl_3$, 282 MHz), with C_6F_6 internal standard (δ -164.9 ppm).

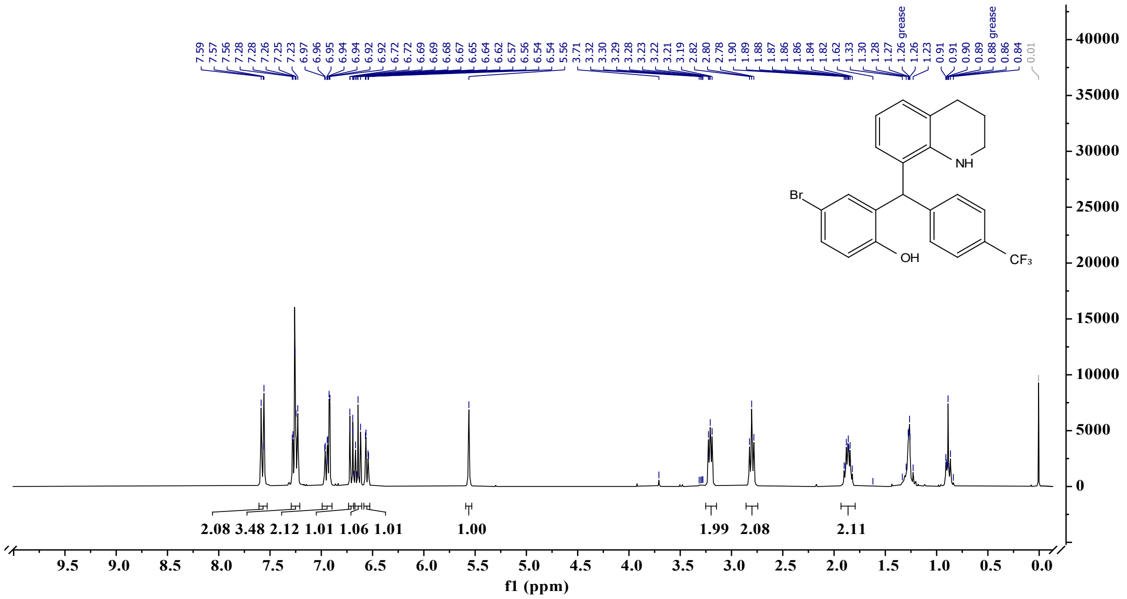

Figure S11:^{1}H NMR of **8b'** ($CDCl_3$, 300 MHz).

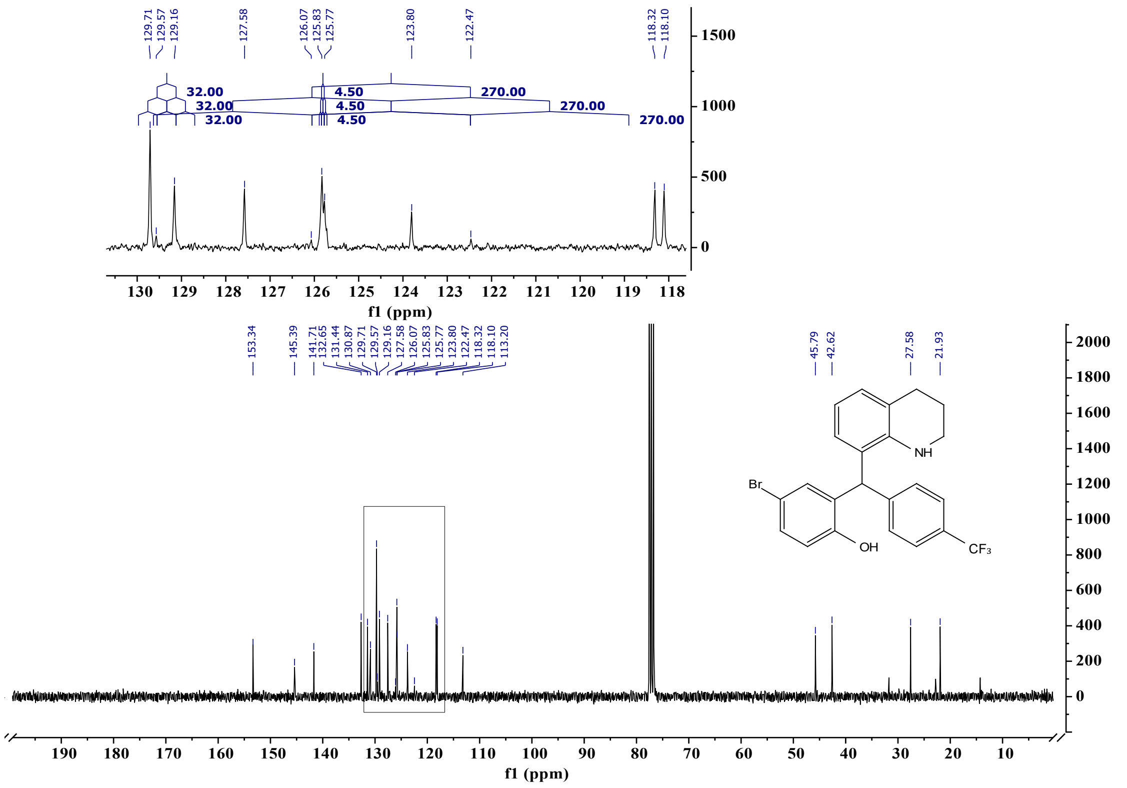

Figure S12:^{13}C NMR of **8b'** ($CDCl_3$, 75 MHz).

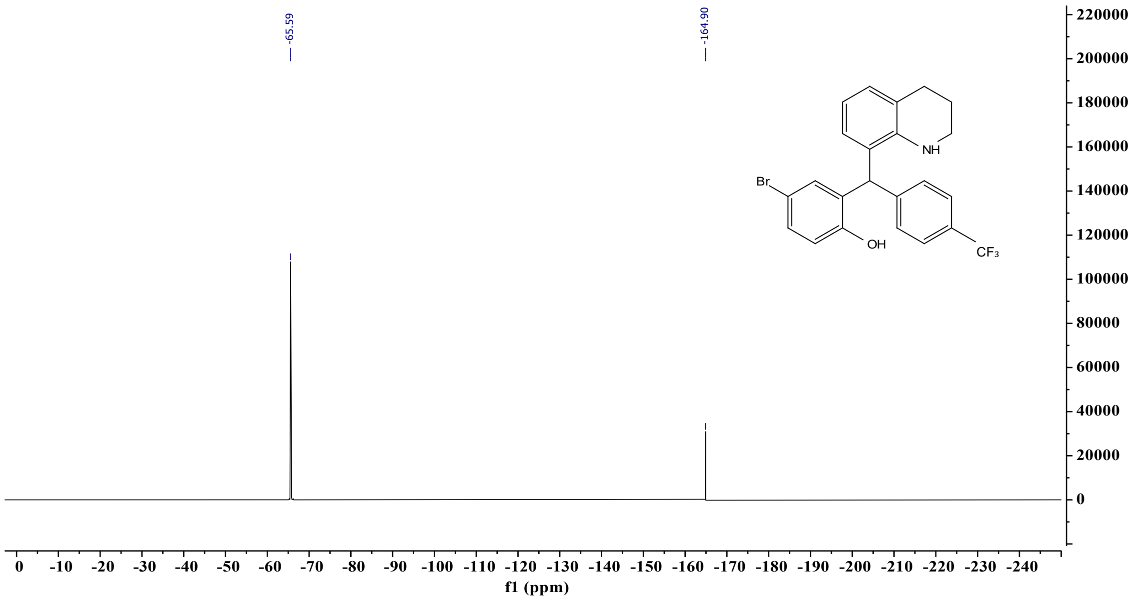

Figure S13:^{19}F NMR of **8b'** ($CDCl_3$, 282 MHz), with C_6F_6 internal standard (δ -164.9 ppm).

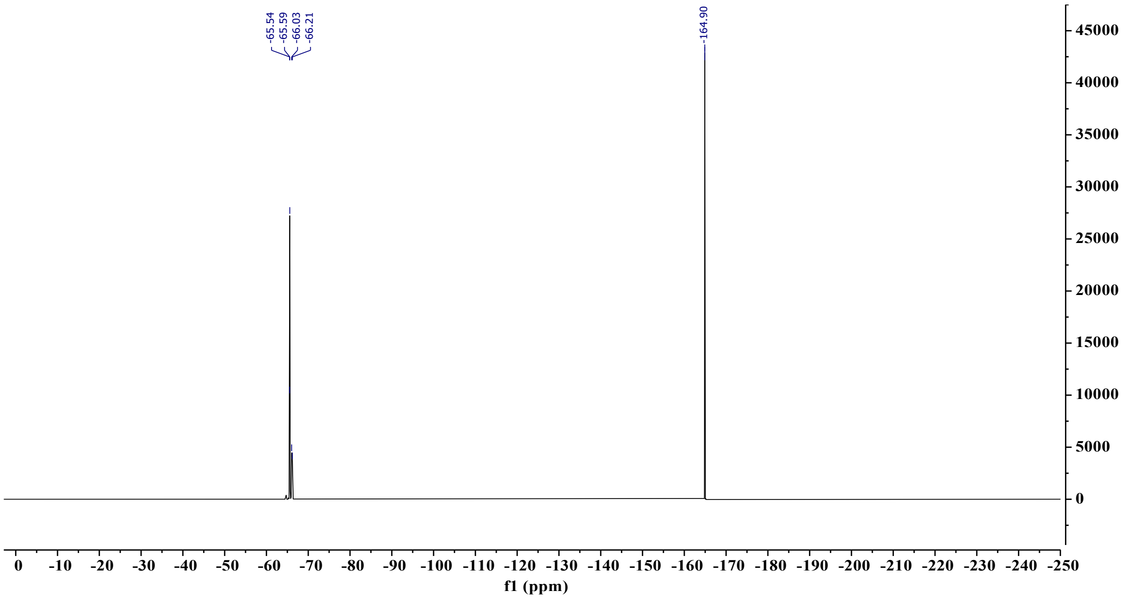

Figure S14:^{19}F NMR crude spectra for reaction with **5b** ($CDCl_3$, 282 MHz), with C_6F_6 internal standard (δ -164.9 ppm).

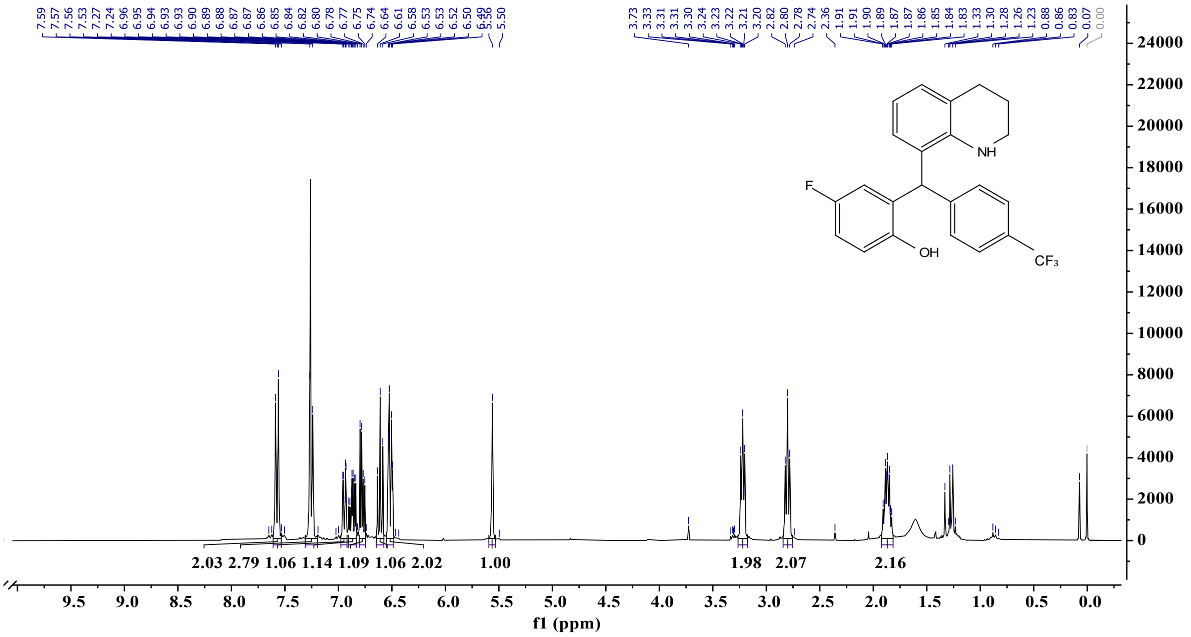

Figure S15:^{1}H NMR of **8c'** ($CDCl_3$, 300 MHz).

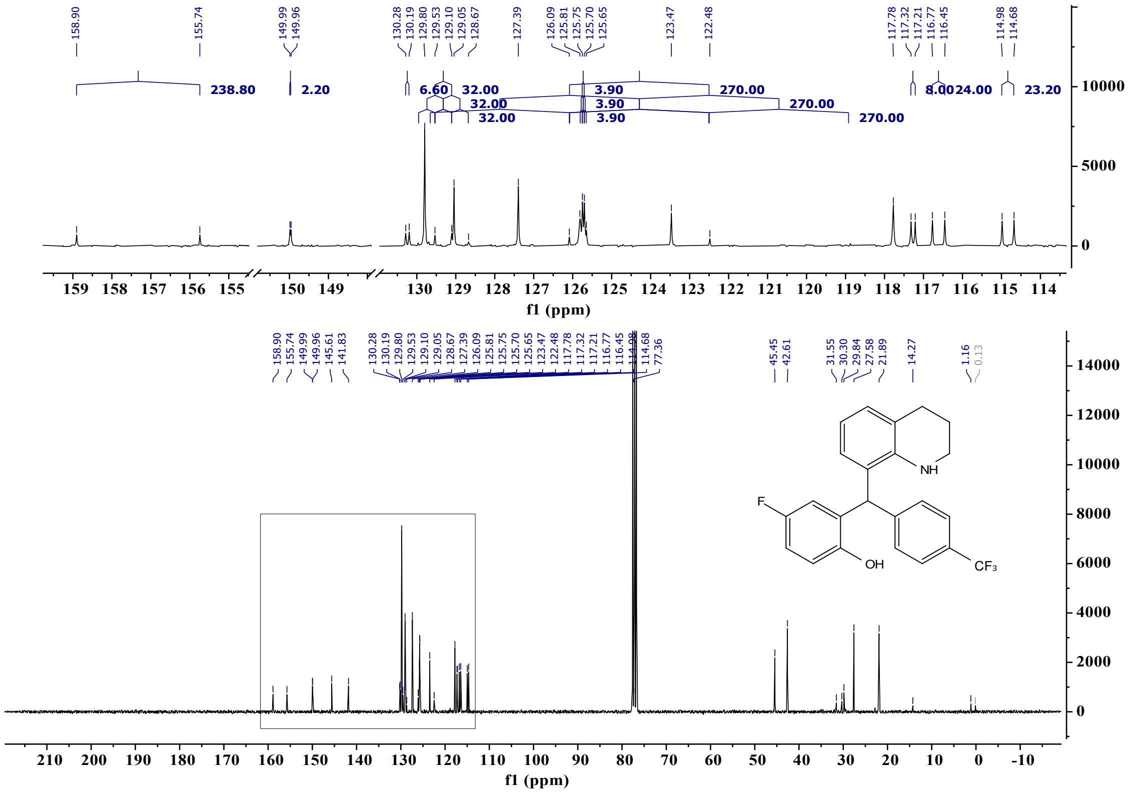

Figure S16:^{13}C NMR of **8c'** ($CDCl_3$, 75 MHz).

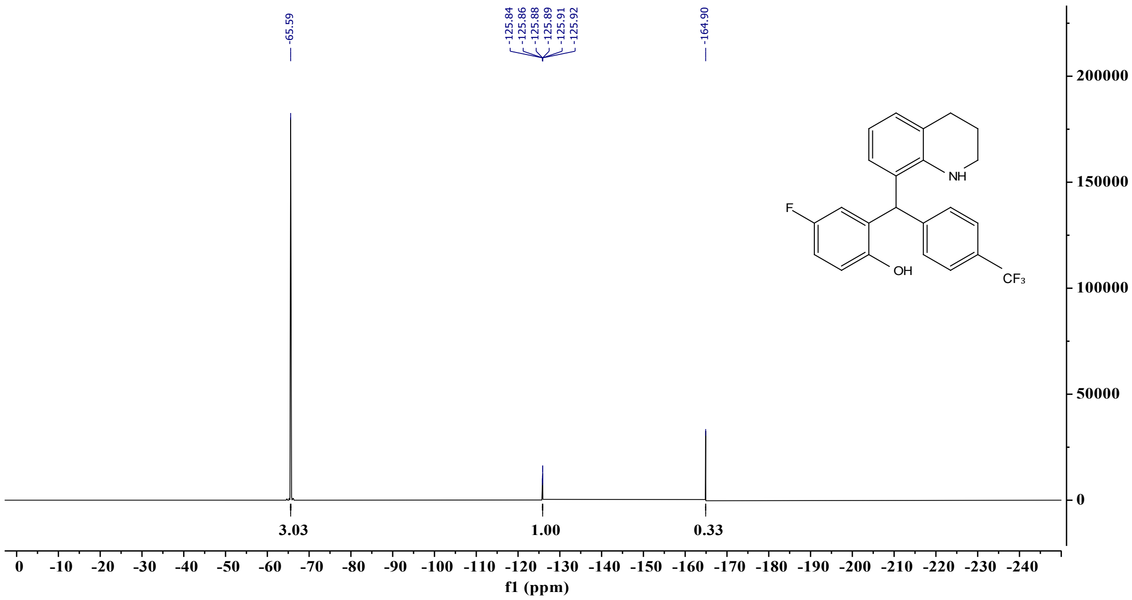

Figure S17:^{19}F NMR of **8c'** ($CDCl_3$, 282 MHz), with C_6F_6 internal standard (δ -164.9 ppm).

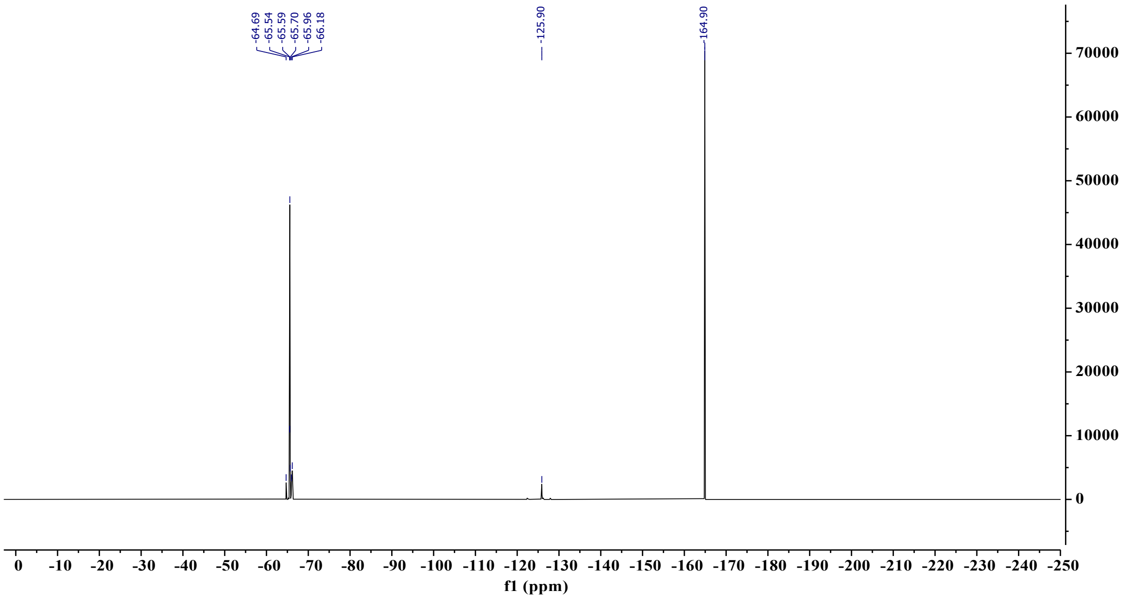

Figure S18:^{19}F NMR crude spectra for reaction with **5c** ($CDCl_3$, 282 MHz), with C_6F_6 internal standard (δ -164.9 ppm).

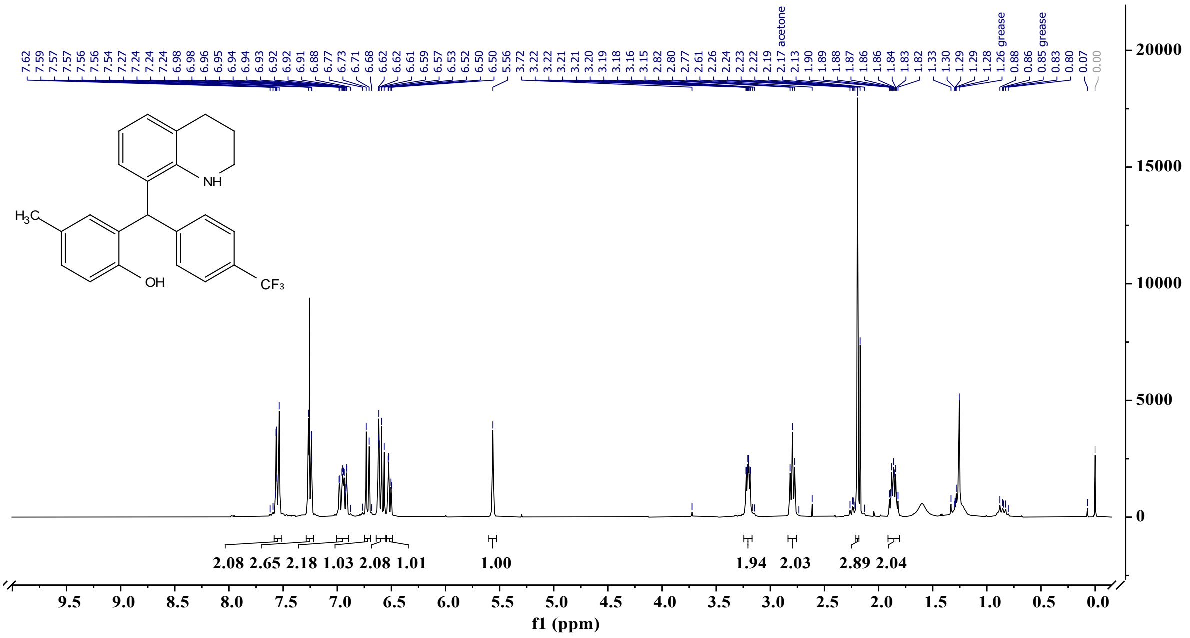

Figure S19:^{1}H NMR of **8d'** ($CDCl_3$, 300 MHz).

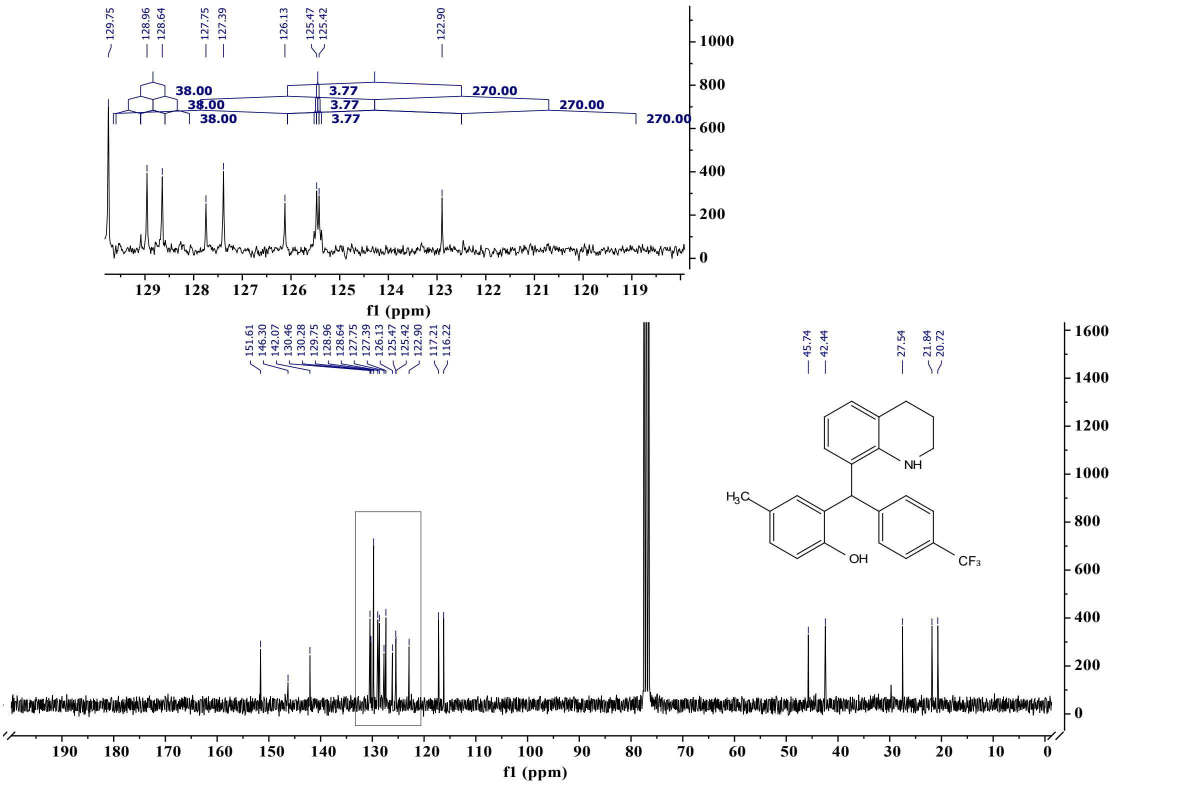

Figure S20:^{13}C NMR of **8d'** ($CDCl_3$, 75 MHz).

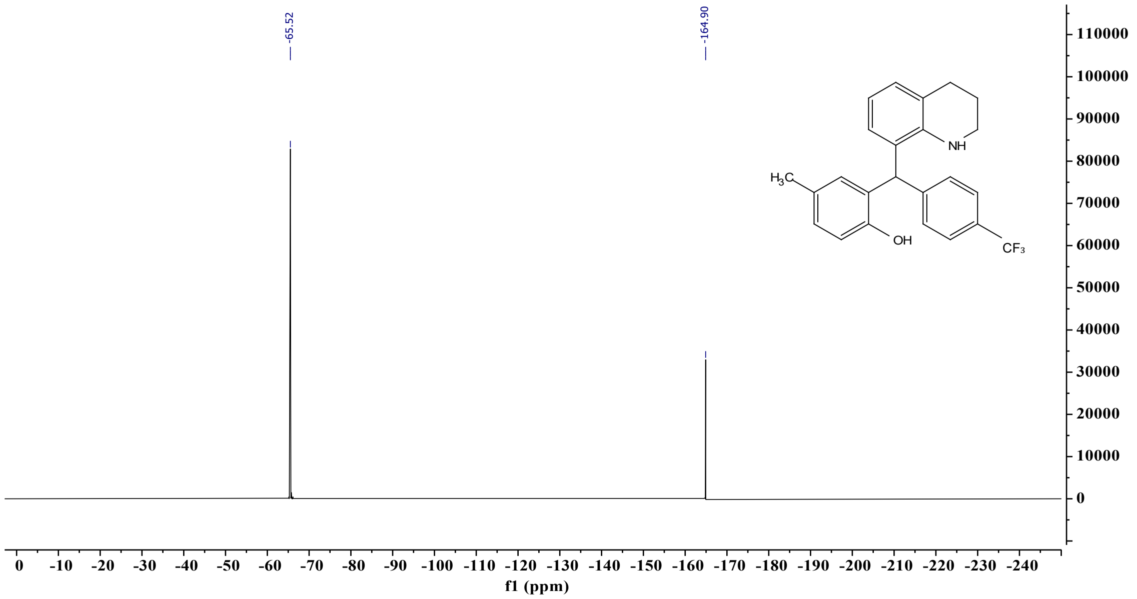

Figure S21:^{19}F NMR of **8d'** ($CDCl_3$, 282 MHz), with C_6F_6 internal standard (δ -164.9 ppm)..

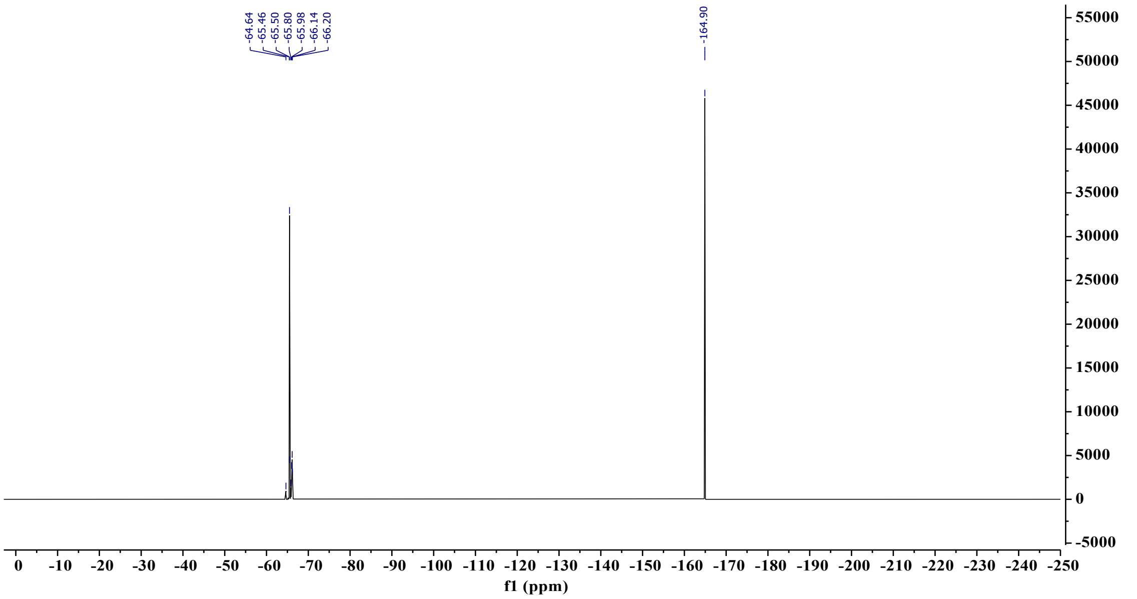

Figure S22:^{19}F NMR crude spectra for reaction with **5d** ($CDCl_3$, 282 MHz), with C_6F_6 internal standard (δ -164.9 ppm).

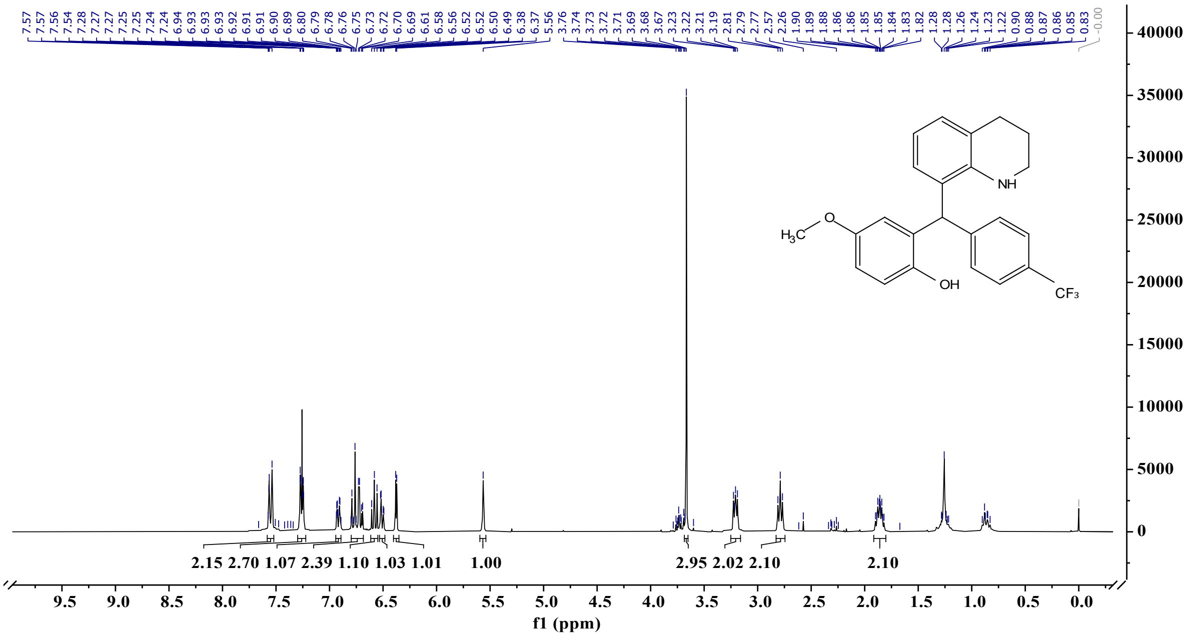

Figure S23:^{1}H NMR of **8e’** ($CDCl_3$, 300 MHz).

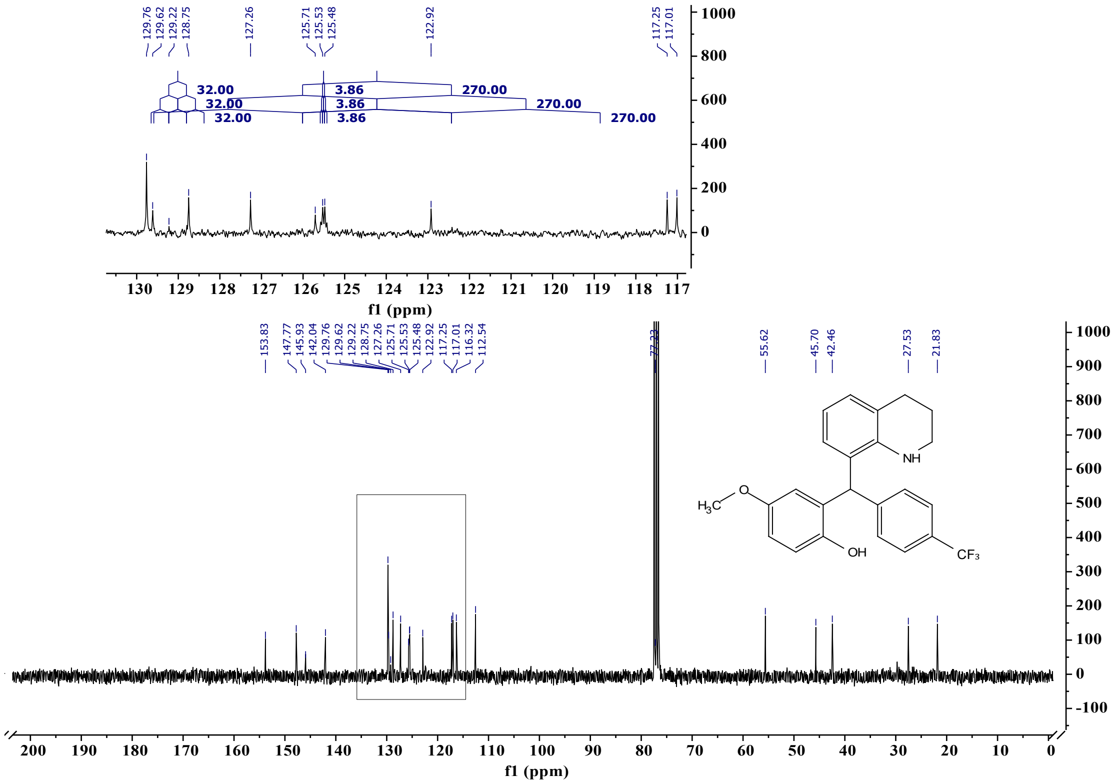

Figure S24:^{13}C NMR of **8e’** ($CDCl_3$, 75 MHz).

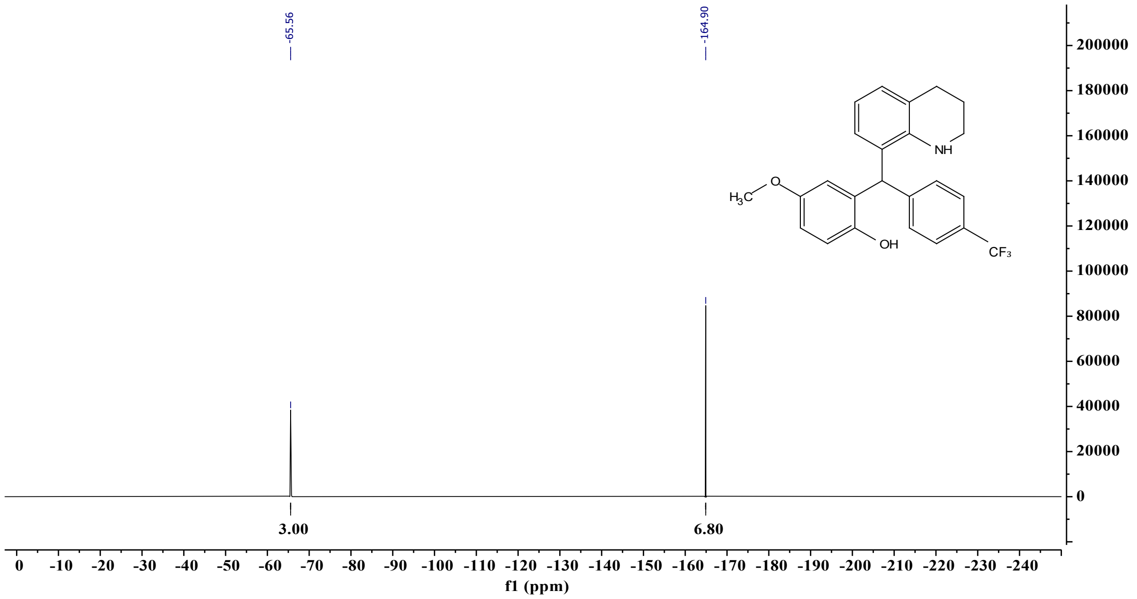

Figure S25:^{19}F NMR of **8e'** ($CDCl_3$, 282 MHz), with C_6F_6 internal standard (δ -164.9 ppm).

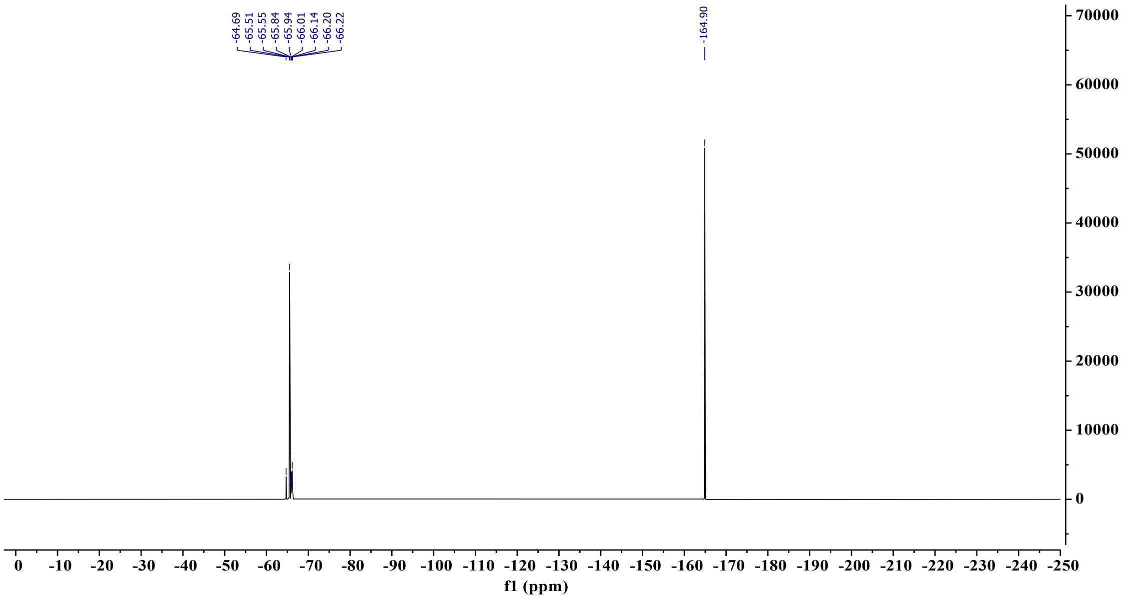

Figure S26:^{19}F NMR crude spectra for reaction with **5e** ($CDCl_3$, 282 MHz), with C_6F_6 internal standard (δ -164.9 ppm).

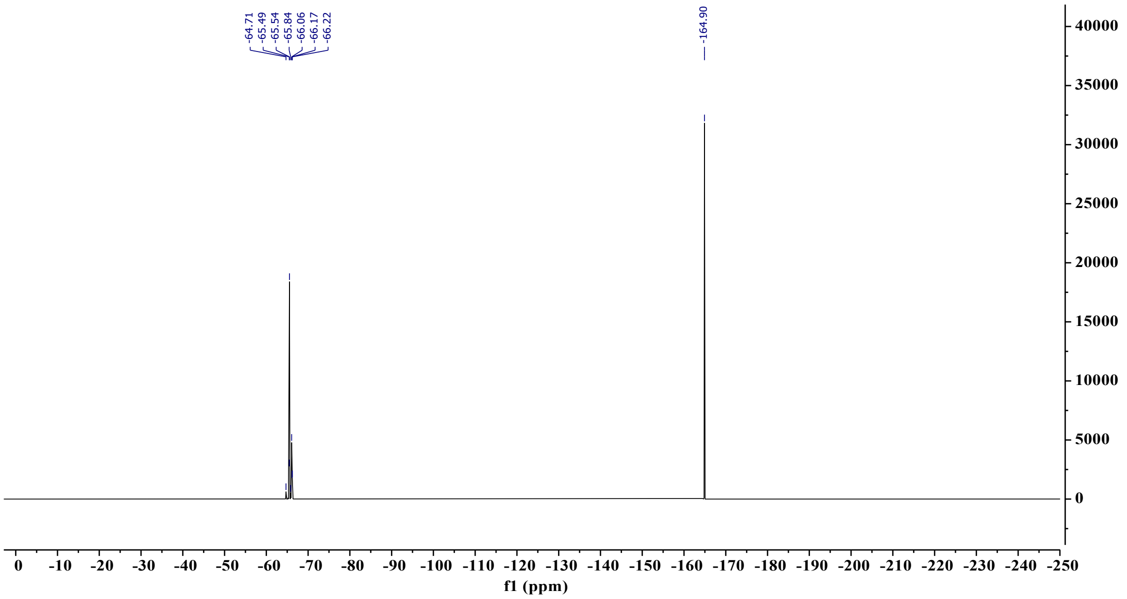

Figure S27:^{19}F NMR crude spectra for reaction with **5f** ($CDCl_3$, 282 MHz), with C_6F_6 internal standard (δ -164.9 ppm).
